# Clinical and Sociodemographic Correlates of Poor Medication Adherence in People With Bipolar Disorder: A Systematic Review and Meta‐Analysis

**DOI:** 10.1111/bdi.70127

**Published:** 2026-06-10

**Authors:** Francesco Bartoli, Daniele Cavaleri, Ilaria Riboldi, Chiara Alessandra Capogrosso, Carlo Bassetti, Marco Broccia, Giorgio Cucchi, Cristina Crocamo, Martha Sajatovic, Giuseppe Carrà

**Affiliations:** ^1^ School of Medicine and Surgery University of Milano‐Bicocca Monza Italy; ^2^ Neurological and Behavioral Outcomes Center University Hospitals Cleveland Medical Center, Case Western Reserve University Cleveland Ohio USA

**Keywords:** adherence, bipolar disorder, clinical correlates, meta‐analysis

## Abstract

**Objectives:**

Poor adherence to psychopharmacological treatment may contribute to relapses in bipolar disorder (BD). We performed a systematic review and meta‐analysis to identify factors associated with poor adherence in BD.

**Methods:**

The protocol was registered in Open Science Framework Registries (https://doi.org/10.17605/OSF.IO/2KZFJ). We searched main electronic databases through March 2025. Random‐effects meta‐analyses were performed to obtain pooled odds ratios (ORs) and standardized mean differences (SMDs) for relevant correlates.

**Results:**

We included 19 studies. Subjects with poor adherence were more likely to be younger (SMD = **−**0.22, 95% CI: **−**0.42–**−**0.02) and to have lower education (SMD = **−**0.34, 95% CI: **−**0.55–**−**0.12), and less likely to be in a relationship (OR = 0.54, 95% CI: 0.34–0.86). Moreover, earlier age at onset (SMD = **−**0.29, 95% CI: **−**0.53–**−**0.04), psychotic features (OR = 1.58, 95% CI: 1.30–1.92), a history of suicide attempts (OR = 1.36, 95% CI: 1.03–1.78), a higher number of manic (SMD = 0.34, 95% CI: 0.08–0.61) and mixed (SMD = 0.16, 95% CI: 0.03–0.28) episodes, and more hospitalizations (SMD = 0.53, 95% CI: 0.32–0.73) all emerged as correlates of poor adherence. Also, cannabis (OR = 2.34, 95% CI: 1.79–3.07) and alcohol use disorders (OR = 1.71, 95% CI: 1.39–2.12), comorbid generalized anxiety disorder (OR = 3.70, 95% CI: 1.90–7.22), and comorbid personality disorders (OR = 5.54, 95% CI: 1.32–23.15) were associated with poor adherence. Finally, poorly adherent individuals had higher global severity (SMD = 0.21, 95% CI: 0.01–0.41), lower insight (SMD = **−**0.74, 95% CI: **−**1.08–**−**0.41), and lower global functioning (SMD = **−**0.60, 95% CI: **−**0.87–**−**0.34). No differences were estimated for other variables.

**Conclusions:**

This meta‐analysis showed that poor adherence in people with BD is associated with specific correlates. Although evidence was generally weak due to small effect sizes, imprecision, inconsistency, and potential publication bias, our findings highlight the importance of strategies to improve adherence.

## 1 Introduction

Bipolar disorder (BD) is a severe and chronic condition, affecting around 2% of the general population [1, 2]. The clinical course is characterized by mood recurrences, in which manic or hypomanic episodes may alternate to depressive episodes [3]. Notwithstanding the availability of several effective therapeutic options for BD, research has shown that different individual and environmental factors are associated with clinical relapses in BD [4]. Among them, poor or suboptimal adherence to psychopharmacological treatment [5, 6], despite attempts based on focused clinical interventions [7, 8], remains common in BD, involving around half of patients [9, 10]. Different reasons can explain this condition, including forgetting to take medications, fear of side effects, disorganized home environments, concern regarding long‐term treatment, and insufficient knowledge regarding BD and its treatment [11]. Recent systematic evidence, though based on limited research, has shown that individual patient's behaviors, lack of social support, and illness‐related factors may impair adherence in people with BD [10]. Nonetheless, poor adherence in BD is a complex phenomenon that may be influenced by several additional factors. Therefore, it is important to identify the full range of determinants of poor adherence [12]. These may involve patient‐ and illness‐related characteristics, such as educational level, marital status, duration of illness, severity of mood episodes, and the presence of comorbid conditions [13, 14, 15]. However, no systematic analyses of socio‐demographic, disease‐related, and individual characteristics influencing adherence in BD are available so far. To address this gap, we performed a systematic review and meta‐analysis of observational studies aimed at identifying correlates of poor adherence in BD. We also assessed the quality of the emerging evidence by evaluating strength, precision, consistency of findings, as well as the risk of publication bias.

## 2 Materials and Methods

### 2.1 Study Design and Protocol

We followed the Meta‐analysis Of Observational Studies in Epidemiology (MOOSE) guidelines to report this systematic review and meta‐analysis [16]. The study protocol was registered on September 2nd, 2024 in Open Science Framework (OSF) registries and updated on March 27th, 2025: https://doi.org/10.17605/OSF.IO/2KZFJ.

### 2.2 Eligibility Criteria

We included any observational studies: (i) involving subjects diagnosed with BD as per standardized diagnostic criteria (e.g., Diagnostic and Statistical Manual of Mental Disorders (DSM), Fourth (DSM‐IV) [17] or Fifth Edition (DSM‐5) [18]; International Classification of Diseases (ICD), 10th revision (ICD‐10) [19] or 11th revision (ICD‐11) [20]); (ii) including samples with a mean age ≥ 18 years; (iii) focusing on adherence to psychopharmacological treatment in BD, considering any drug type/class used to treat the disorder (mood stabilizers, oral or long‐acting antipsychotics, antidepressants); (iv) distinguishing good and poor adherence according to a clearly defined measurement, either by clinical evaluation/rating or with objective assessments (e.g., blood, urine, or hair concentration) methods, to enhance reliability and avoid self‐report bias; (v) reporting on at least one socio‐demographic (e.g., age, sex, socio‐economic status) or clinical (e.g., symptom severity, comorbidities, treatment factors) correlate of treatment adherence; (vi) including at least 10 individuals in each group.

We excluded studies: (i) not exploring exclusively subjects with BD (e.g., mixed psychiatric populations without separate data for BD); (ii) investigating samples with a mean age < 18 years, unless there was a subgroup ≥ 18 years old and it was reported on separately; (iii) focusing on non‐pharmacological treatments (e.g., psychoeducation, psychotherapy, lifestyle interventions) without analysis of psychopharmacotherapy adherence; (iv) not reporting on adherence or lacking a clear definition/measurement of adherence to psychopharmacotherapy treatment; (v) using exclusively measures of adherence, such as self‐report questionnaires, drug dispensing assessment, or medication possession ratio, as these methods are susceptible to self‐report bias and do not provide direct or clinically verifiable evidence of actual drug intake; (vi) reporting adherence as continuous scores without clearly separating high vs. low adherence; (vii) not assessing the association between socio‐demographic or clinical factors and adherence; (viii) published before the release of DSM‐IV (1994); (ix) case reports and case series; (x) scientific reports not undergoing peer‐review process, such as conference abstracts, dissertations/theses, and gray literature.

To reduce the risk of selective reporting bias, two investigators (F.B. and D.C.) contacted the corresponding authors of studies that were not eligible—e.g., because participants were not classified into distinct categories of poor vs. good adherence—in order to obtain additional unpublished data for inclusion in our meta‐analyses.

Moreover, in order to avoid duplicate results, we excluded multiple publications on the same sample, including only the study that provided the most comprehensive amount of information.

### 2.3 Article Search and Screening

We searched MEDLINE (via Ovid), Embase (via Embase), and APA PsycInfo (via ProQuest) for articles indexed from 1994 up to March 27th, 2025, without any language restrictions. We used the following search phrases adapted for each database: (i) MEDLINE (via Ovid): “(bipolar OR mania OR manic).ti,ab AND (adherence OR compliance).ti,ab”; (ii) Embase (via Embase): “(bipolar:ti,ab OR mania:ti,ab OR manic:ti,ab) AND (adherence:ti,ab OR compliance:ti,ab)”; (iii) APA PsycInfo (via ProQuest): “TI,AB(bipolar OR mania OR manic) AND TI,AB(adherence OR compliance)”.

An additional manual search of studies included in relevant reviews [10, 12, 21, 22] was carried out to check for further potentially eligible studies. References were managed using Zotero 7 (https://www.zotero.org/). After the preliminary screening based on titles and abstracts, full texts were retrieved to assess the final eligibility of studies. These procedures were completed by three authors (F.B., C.B., and M.B.) independently, and reasons for exclusion after full‐text review were recorded. Disagreements concerning suitability for inclusion were resolved by discussion and consensus involving all authors.

### 2.4 Data Extraction

Data were extracted and inserted into standard .xls templates by six authors (D.C., I.R., C.A.C., C.B., M.B., and G.Cu.) independently and subsequently blindly cross‐checked for accuracy.

Key article information and sample characteristics (including year of publication, country, setting, inclusion and exclusion criteria, sample size, mean age, sex proportion, and assessment of adherence to psychopharmacological treatment) were collected from all eligible studies.

Data on socio‐demographic and clinical correlates of poor adherence to psychopharmacological treatment were extracted.

### 2.5 Data Analysis

In order to compare, as for relevant correlates, people with BD who had poor or good medication adherence according to each included paper's criteria and cutoff, random‐effects meta‐analyses were conducted for variables with data available from at least three different studies. A *p*‐value below 0.05 was the chosen threshold for statistical significance. We used odds ratio (OR) and Hedges' g (standardized mean difference—SMD), with their 95% CI, for categorical and continuous variables, respectively. Odds ratios were estimated comparing the number of positive cases for a tested variable in non‐adherent vs. adherent groups. Hedges' g values were estimated comparing the reported means and standard deviations (SD) of continuous variables in each group. When data were reported as medians with minimum/maximum values or interquartile ranges, we converted them to the corresponding means and SDs using the recommended formulas for conversion [23, 24]. When means and/or SDs could not be obtained, we used *Practical Meta Analysis Effect Size Calculator* [25] to convert available data into effect sizes. Heterogeneity across studies was evaluated according to standard cut‐offs for I^2^ statistics to measure inconsistency of meta‐analyses [26]. Publication bias was assessed using Egger's test for meta‐analyses with data available from at least 10 studies [27]. To evaluate the magnitude and precision of the effects, each OR of categorical variables was converted into SMD dividing the relevant ln(OR) by 1.81 [28]. Based on conventional cut‐offs [29], we used the following intervals to interpret the magnitude of the effects: very small: < 0.2; small: ≥ 0.2 to < 0.35; small‐to‐medium: ≥ 0.35 to < 0.50; medium: ≥ 0.50 to < 0.65; medium‐to‐large: ≥ 0.65–0.80; large: ≥ 0.80. Data analyses were performed using Stata statistical software, release 18 [30].

### 2.6 Grading of the Evidence

Following an approach similarly used in recent meta‐analyses [31], we used GRADE (Grading of Recommendations, Assessment, Development, and Evaluations) items [32], adapted for observational studies, to classify the quality of evidence as high, moderate, low, or very low. Accordingly, for each variable showing a statistically significant pooled estimate (*p* < 0.05), we evaluated consistency and precision of meta‐analytic findings, the risk of publication bias, as well as the magnitude of the effect estimate.

First, we assessed the ‘*consistency*’ of findings according to the I^2^ value. We downgraded by one level the quality of evidence if inconsistency was estimated (I^2^ ≥ 50%).

Second, we evaluated the ‘*precision*’ of findings by checking the width of the equivalent effect size's 95% CI. We downgraded the quality of evidence by one level if (i) 95% CI width of the equivalent effect size was ≥ 0.40.

Third, we assessed the risk of ‘*publication bias*’, downgrading the quality of evidence by one level if (i) meta‐analyses included less than 10 studies or (ii) the Egger's test *p* < 0.10 for meta‐analyses including at least 10 studies.

Finally, we evaluated the ‘*magnitude of the effect*’, upgrading the quality of evidence by one level if the effect size was large (SMD ≥ 0.80) or downgrading by one level if it was small (SMD < 0.35).

## 3 Results

### 3.1 Study Selection

The systematic search on relevant databases generated 6088 records, namely 1665 from MEDLINE, 3201 from EMBASE, and 1222 from APA PsycInfo. After deduplication, 3351 articles were retained for screening. In addition, 79 studies were retrieved from the reference lists of four reviews [10, 12, 21, 22]. After screening by titles and abstracts, 211 studies from databases and 17 from reviews were identified as potentially eligible. Following the full text screening, 19 studies met the eligibility criteria and were included in the meta‐analysis [15, 33, 34, 35, 36, 37, 38, 39, 40, 41, 42, 43, 44, 45, 46, 47, 48, 49, 50].

We obtained additional, unpublished data for three studies [33, 35, 48].

**FIGURE 1:**
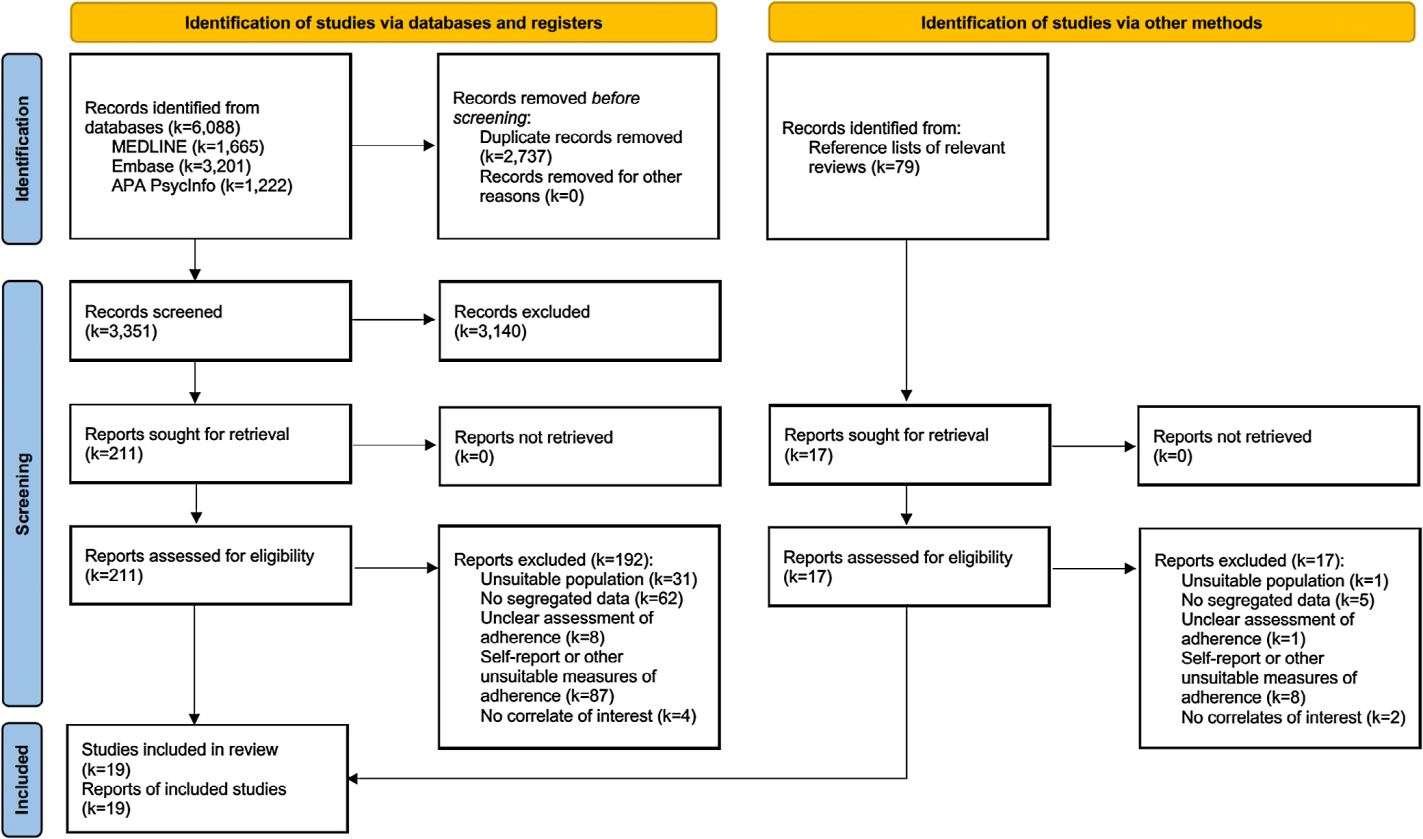
Flow chart of study inclusion process. *Source:* Page MJ, et al. BMJ 2021; 372: N71. Doi: 10.1136/bmj.n71.

A flow chart with screening details, study selection process, and reasons for exclusion is presented in Figure 1.

### 3.2 Study Characteristics

Studies were published between 1997 [44] and 2023 [33, 35] (Figure S1).

The sample sizes varied between 44 [39] and 2205 [34].

The populations studied varied in age (mean values between 27.1 ± 7.2 and 52.5 ± 14.8 years) and gender distribution (male participants ranging from 20.2% to 71.1%).

The majority of studies (k = 17) were conducted in Europe, with Spain contributing the highest number of studies (k = 9) [15, 36, 39, 40, 41, 46, 47, 48, 49] followed by Italy (k = 4) [33, 35, 38, 50].

The assessments of adherence included clinical evaluation (k = 9) [33, 34, 35, 38, 39, 41, 45, 47, 50], measurement of blood concentrations of psychotropic drugs (k = 4) [37, 42, 43, 49], or both (k = 6) [15, 36, 40, 44, 46, 48].

Poor adherence rates widely varied across studies, ranging from 13.9% to 69.3%, reflecting variability in samples, settings, and assessment methods.

Study characteristics are reported in Table 1.

**TABLE 1:** Characteristics of the included studies.

Author(s), year	Country	Study design	Setting	Sample size *N*	Age (years) mean ± SD	Male gender *N* (%)	Assessment of adherence	Poor adherence *N (%)*
Bartoli et al., 2023	Italy	mirror‐image	inpatient and outpatient	71	46.2 ± 13.4	38 (53.5%)	Compliance Rating Scale	27 (38.0%)
Bellivier et al., 2011	France	prospective	inpatient and outpatient	2205	44.7 ± 13.3*	932 (42.0%)*	Clinician evaluation	605 (27.4%)
Benatti et al., 2023	Italy	cross‐sectional	inpatient and outpatient	108	50.3 ± 16.2	33 (30.6%)	Compliance Rating Scale	33 (30.6%)
Colom et al., 2000	Spain	prospective	outpatient	200	43.5 ± 15.3	88 (44.0%)	1. Blood concentrations of mood stabilizers during the previous 2 years 2. Clinical interview to patient and relatives/caregivers	79 (39.5%)
Darling et al., 2008	USA	case–control	outpatient	100	42.8**	40 (40.0%)	1. Blood concentrations of mood stabilizers during the previous 6 months (2. Self‐report)	50 (50.0%)
Fornaro et al., 2013	Italy	cross‐sectional	outpatient	220	39.0 ± 12.5	91 (41.3%)	1. Compliance Rating Scale (2. Self‐report)	49 (22.3%)
García et al., 2019	Spain	prospective	outpatient***	44	27.1 ± 7.2	31 (71.1%)	1. Clinician evaluation (2. Self‐report)	16 (36.4%)
González‐Pinto et al., 2006	Spain	prospective	inpatient	72	52.5 ± 14.8	33 (45.8%)	1. Blood concentrations of lithium (bimonthly for 5 years) 2. Semi‐structured clinical evaluation with patient and relatives/caregivers	16 (22.2%)
González‐Pinto et al., 2010	Spain	prospective	inpatient and outpatient	1831	44.8 ± 13.2	787 (43.0%)	Clinician evaluation	429 (23.4%)
Gutiérrez‐Rojas et al., 2020	Spain	cross‐sectional	inpatient and outpatient	108	48.2 ± 14.1	33 (30.6%)	1. Blood concentrations of mood stabilizers 2. Clinical interview to patient and relatives/caregivers	18 (16.7%)
Jónsdóttir et al., 2013	Norway	cross‐sectional	outpatient	101	38.8 ± 12.1	41 (40.6%)	1. Blood concentrations of psychotropic drugs and concentration/dose ratio (2. Self‐report)	15 (13.9%)
Karadağ et al., 2019	Turkey	cross‐sectional	outpatient	117	38.9 ± 11.3	48 (41.0%)	1. Blood concentrations of mood stabilizers (2. Self‐report)	19 (16.2%)
Keck et al., 1997	USA	prospective	inpatient	140	28.9 ± 6.5	71 (50.7%)	1. Blood concentrations of mood stabilizers 2. Clinical report (3. Self‐report by patient and relatives/caregivers)	43 (30.7%)
Kutzelnigg et al., 2014	Austria	prospective	outpatient	891	43.3 ± 12.8	378 (42.4%)	Clinician evaluation	297 (33.3%)
Martinez‐Aran et al., 2009	Spain	cross‐sectional	inpatient	103	39.7 ± 10.7	47 (45.6%)	1. Blood concentrations of mood stabilizers during the previous 2 years 2. Clinical interview to patient and relatives/caregivers	42 (40.7%)
Montes et al., 2013	Spain	cross‐sectional	outpatient	303	45.9 ± 12.8	122 (40.3%)	1. Compliance Rating Scale (2. Self‐report)	210 (69.3%)
Murru et al., 2013	Spain	cross‐sectional	outpatient	150	Not reported	73 (48.7%)	1. Blood concentrations of mood stabilizers 2. Clinical interview to patient and relatives/caregivers	49 (32.7%)
Navarro et al., 2016	Spain	cross‐sectional	outpatient	76	49.2 ± 11.3	28 (36.8%)	1. Blood concentrations of mood stabilizers (2. Self‐report)	28 (36.8%)
Niolu et al., 2015	Italy	prospective	inpatient	89	Not reported	18 (20.2%)	1. Clinician evaluation (2. Self‐report by patient and relatives/caregivers)	33 (37.1%)

* For the total sample.

** SD not reported.

*** First recruited in an inpatient setting.

### 3.3 Correlates of Poor Adherence to Treatment in People With Bipolar Disorder

#### 3.3.1 Socio‐Demographic Correlates

The meta‐analysis showed that subjects with poor adherence to treatment were more likely to be younger (k = 14; *N* = 4220; SMD = **−**0.22, 95% CI: −0.42 to −0.02, *p* = 0.031; I^2^ = 83.5%) (Figure S2), with no evidence of publication bias (Egger's *p* = 0.430). No differences between subjects with poor and those with good adherence in terms of gender emerged (k = 16; *N* = 4517; OR = 1.02, 95% CI: 0.81 to 1.28, *p* = 0.867; I^2^ = 47.8%) (Figure S3), without any evidence of publication bias (Egger's *p* = 0.837). The relevant meta‐regression analysis showed no influence of age (*p* = 0.511).

In addition, subjects with poor adherence to treatment had fewer years of education (k = 9; *N* = 2675; SMD = **−**0.34, 95% CI: −0.55 to −0.12, *p* = 0.002; I^2^ = 60.2%) (Figure S4) and were less likely to reach higher educational levels (k = 5; *N* = 2249; OR = 0.71, 95% CI: 0.55 to 0.92, *p* = 0.010; I^2^ = 0%) (Figure S5) as compared with their adherent counterpart.

Also, subjects with poor adherence to treatment were less likely to be in a relationship (k = 9; *N* = 1056; OR = 0.54, 95% CI: 0.34 to 0.86, *p* = 0.009; I^2^ = 56.0%) (Figure S6).

No statistically significant differences in rates of unemployment (k = 8; *N* = 1730; OR = 1.51, 95% CI: 0.83 to 2.76, *p* = 0.177; I^2^ = 70.0%) (Figure S7) as well as in the likelihood of living alone (k = 6; *N* = 814; OR = 0.89, 95% CI: 0.52 to 1.52, *p* = 0.664; I^2^ = 33.7%) (Figure S8) were shown.

#### 3.3.2 Clinical Correlates

Poorly adherent subjects had a younger age at onset (k = 9; *N* = 2761; SMD = **−**0.29, 95% CI: −0.53 to −0.04, *p* = 0.023; I^2^ = 79.2%) (Figure S9), although no significant differences in illness duration were found (k = 6; *N* = 739; SMD = **−**0.12, 95% CI: −0.28 to 0.05, *p* = 0.168; I^2^ = 9.2%) (Figure S10).

Poorly adherent subjects were more likely to show psychotic features (k = 4; *N* = 2210; OR = 1.58, 95% CI: 1.30 to 1.92, *p* < 0.001; I^2^ = 0%) (Figure S11) and to have a history of suicide attempts (k = 7; *N* = 4609; OR = 1.36, 95% CI: 1.03 to 1.78, *p* = 0.030; I^2^ = 37.0%) (Figure S12). On the other hand, no statistically significant differences in the likelihood of having a family history of mood disorders (k = 4; *N* = 419; OR = 0.79, 95% CI: 0.53 to 1.18, *p* = 0.249; I^2^ = 0%) (Figure S13), a diagnosis of BD‐I (k = 3; *N* = 606; OR = 1.28, 95% CI: 0.51 to 3.21, *p* = 0.599; I^2^ = 80.1%) (Figure S14), and a rapid cycling course (k = 4; *N* = 2368; OR = 2.40, 95% CI: 0.58 to 9.89, *p* = 0.705; I^2^ = 89.3%) (Figure S15) emerged between the two groups.

Individuals with poor adherence were more likely to report more previous manic (k = 6; *N* = 2432; SMD = 0.34, 95% CI: 0.08 to 0.61, *p* = 0.011; I^2^ = 79.8%) (Figure S16) and mixed episodes (k = 6; *N* = 2432; SMD = 0.16, 95% CI: 0.03 to 0.28, *p* = 0.013; I^2^ = 9.1%) (Figure S17). However, no differences in the number of total (k = 6; *N* = 718; SMD = 0.33, 95% CI: −0.07 to 0.73, *p* = 0.106; I^2^ = 82.8%) (Figure S18) or depressive (k = 6; *N* = 2432; SMD = 0.08, 95% CI: −0.13 to 0.30, *p* = 0.429; I^2^ = 68.0%) (Figure S19) relapses were found. Poorly adherent subjects had also a higher number of previous hospitalizations (k = 4; *N* = 413; SMD = 0.53, 95% CI: 0.32 to 0.73, *p* < 0.001; I^2^ = 0%) (Figure S20) than those with good adherence to treatment, although no statistically significant difference was found between the two groups in the likelihood of having had at least one previous hospitalization (k = 4; *N* = 2086; OR = 1.17, 95% CI: 0.66 to 2.08, *p* = 0.583; I^2^ = 51.1%) (Figure S21).

Poorly adherent subjects were more likely to have a substance use disorder (k = 10; *N* = 1159; OR = 3.32, 95% CI: 1.94 to 5.70, *p* < 0.001; I^2^ = 58.7%) (Figure S22), with no evidence of publication bias (Egger's *p* = 0.236). More specifically, poorly adherent individuals were more likely to have a cannabis use disorder (k = 4; *N* = 2281; OR = 2.34, 95% CI: 1.79 to 3.07, *p* < 0.001; I^2^ = 0%) (Figure S23) and an alcohol use disorder (k = 5; *N* = 2352; OR = 1.71, 95% CI: 1.39 to 2.12, *p* < 0.001; I^2^ = 0%) (Figure S24).

Poorly adherent subjects were more likely to have also comorbid generalized anxiety disorder (k = 3; *N* = 375; OR = 3.70, 95% CI: 1.90 to 7.22, *p* < 0.001; I^2^ = 3.9%) (Figure S25) and personality disorder (k = 4; *N* = 605; OR = 5.54, 95% CI: 1.32 to 23.15, *p* = 0.019; I^2^ = 84.4%) (Figure S26).

#### 3.3.3 Treatment Correlates

No differences were found between the two groups as for the likelihood of being prescribed with polytherapy (k = 3; *N* = 347; OR = 0.95, 95% CI: 0.42 to 2.16, *p* = 0.905; I^2^ = 49.4%) (Figure S27).

Also, our meta‐analyses did not show any differences in the likelihood of being on lithium (k = 8; *N* = 2706; OR = 1.25, 95% CI: 0.83 to 1.88, *p* = 0.293; I^2^ = 51.0%) (Figure S28), valproate (k = 6; *N* = 2463; OR = 1.28, 95% CI: 0.69 to 2.38, *p* = 0.428; I^2^ = 68.6%) (Figure S29), carbamazepine (k = 4; *N* = 544; OR = 1.32, 95% CI: 0.90 to 1.94, *p* = 0.157; I^2^ = 0%) (Figure S30), antidepressants (k = 6; *N* = 2386; OR = 1.13, 95% CI: 0.90 to 1.41, *p* = 0.312; I^2^ = 0%) (Figure S31), and antipsychotics (k = 3; *N* = 250; OR = 1.40, 95% CI: 0.78 to 2.50, *p* = 0.258; I^2^ = 16.5%) (Figure S32) – regardless of whether of first‐ (k = 4; *N* = 2239; OR = 1.00, 95% CI: 0.72 to 1.39, *p* = 0.980; I^2^ = 18.6%) (Figure S33) or second‐generation (k = 5; *N* = 2463; OR = 1.75, 95% CI: 0.78 to 3.89, *p* = 0.174; I^2^ = 87.5%) (Figure S34) – between subjects with poor and good adherence.

#### 3.3.4 Other Correlates

Poorly adherent subjects had higher global severity (k = 5; *N* = 3321; SMD = 0.21, 95% CI: 0.01 to 0.41, *p* = 0.042; I^2^ = 74.2%) (Figure S35) than those with good adherence.

Moreover, poorly adherent individuals showed lower insight (k = 4; *N* = 1219; SMD = **−**0.74, 95% CI: −1.08 to −0.41, *p* < 0.001; I^2^ = 61.8%) (Figure S36) and global functioning (k = 4; *N* = 599; SMD = **−**0.60, 95% CI: −0.87 to −0.34, *p* < 0.001; I^2^ = 45.3%) (Figure S37).

No statistically significant differences in terms of general medical comorbidities (k = 4; *N* = 427; OR = 0.70, 95% CI: 0.34 to 1.43, *p* = 0.324; I^2^ = 41.6%) (Figure S38) between the two groups were found.

Finally, no significant differences in drug attitude scores (k = 3; *N* = 1038; SMD = **−**0.59, 95% CI: −1.19 to 0.00, *p* = 0.052; I^2^ = 86.8%) (Figure S39) were observed.

The forest plots of the meta‐analyses are reported in Supporting Information.

A summary of findings is provided in Table 2.

**TABLE 2:** Correlates of poor adherence to treatment in people with bipolar disorder: Meta‐analyses.

Correlate	*k*	*N*	Effect size [95% CI]	*p*	I^2^
Age	14	4220	SMD = **−**0.22 [−0.42 to −0.02]	0.031	83.5%
Male gender	16	4517	OR = 1.02 [0.81 to 1.28]	0.867	47.8%
Years of education	9	2675	SMD = **−**0.34 [−0.55 to −0.12]	0.002	60.2%
Higher education	5	2249	OR = 0.71 [0.55 to 0.92]	0.010	0%
Unemployed	8	1730	OR = 1.51 [0.83 to 2.76]	0.177	70.0%
Living alone	6	814	OR = 0.89 [0.52 to 1.52]	0.664	33.7%
In a relationship	9	1056	OR = 0.54 [0.34 to 0.86]	0.009	56.0%
Age at onset	9	2761	SMD = **−**0.29 [−0.53 to −0.04]	0.023	79.2%
Duration of illness	6	739	SMD = **−**0.12 [−0.28 to 0.05]	0.168	9.2%
Diagnosis of bipolar disorder type I	3	606	OR = 1.28 [0.51 to 3.21]	0.599	80.1%
Family history of mood disorders	4	419	OR = 0.79 [0.53 to 1.18]	0.249	0%
Psychotic features	4	2210	OR = 1.58 [1.30 to 1.92]	< 0.001	0%
Rapid cycling	4	2368	OR = 2.40 [0.58 to 9.89]	0.705	89.3%
History of suicide attempts	7	4609	OR = 1.36 [1.03 to 1.78]	0.030	37.0%
Total number of previous mood episodes	6	718	SMD = 0.33 [−0.07 to 0.73]	0.106	82.8%
Number of previous manic episodes	6	2432	SMD = 0.34 [0.08 to 0.61]	0.011	79.8%
Number of previous mixed episodes	6	2432	SMD = 0.16 [0.03 to 0.28]	0.013	9.1%
Number of previous depressive episodes	6	2432	SMD = 0.08 [−0.13 to 0.30]	0.429	68.0%
History of hospitalization	4	2086	OR = 1.17 [0.66 to 2.08]	0.583	51.1%
Number of previous hospitalizations	4	413	SMD = 0.53 [0.32 to 0.73]	< 0.001	0%
Substance use disorder	10	1159	OR = 3.32 [1.94 to 5.70]	< 0.001	58.7%
Cannabis use disorder	4	2281	OR = 2.34 [1.79 to 3.07]	< 0.001	0%
Alcohol use disorder	5	2352	OR = 1.71 [1.39 to 2.12]	< 0.001	0%
Comorbid generalized anxiety disorder	3	375	OR = 3.70 [1.90 to 7.22]	< 0.001	3.9%
Comorbid personality disorder	4	605	OR = 5.54 [1.32 to 23.15]	0.019	84.4%
Polytherapy	3	347	OR = 0.95 [0.42 to 2.16]	0.905	49.4%
Lithium prescription	8	2706	OR = 1.25 [0.83 to 1.88]	0.293	51.0%
Valproate prescription	6	2463	OR = 1.28 [0.69 to 2.38]	0.428	68.6%
Carbamazepine prescription	4	544	OR = 1.32 [0.90 to 1.94]	0.157	0%
Antidepressants prescription	6	2386	OR = 1.13 [0.90 to 1.41]	0.312	0%
Antipsychotics prescription	3	250	OR = 1.40 [0.78 to 2.50]	0.258	16.5%
First‐generation antipsychotics prescription	4	2239	OR = 1.00 [0.72 to 1.39]	0.980	18.6%
Second‐generation antipsychotics prescription	5	2463	OR = 1.75 [0.78 to 3.89]	0.174	87.5%
Global severity	5	3321	SMD = 0.21 [0.01 to 0.41]	0.042	74.2%
Insight	4	1219	SMD = **−**0.74 [−1.08 to −0.41]	< 0.001	61.8%
Global functioning	4	599	SMD = **−**0.60 [−0.87 to −0.34]	< 0.001	45.3%
General medical comorbidities	4	427	OR = 0.70 [0.34 to 1.43]	0.324	41.6%
Drug attitude scores	3	1038	SMD = **−**0.59 [−1.19 to 0.00]	0.052	86.8%

Abbreviations: 95% CI, 95% confidence interval; *k*, number of studies; *N*, number of subjects; OR, odds ratio; SMD, standardized mean difference.

### 3.4 Grading of the Evidence

Following evidence grading criteria, including effect size, precision, consistency, and publication bias, no body of evidence could be deemed of high quality. The evidence of higher rates of cannabis use disorder in poorly adherent patients was considered of moderate quality. All evidence on the remaining correlates was graded as of low or very low quality.

The overall assessment of the quality of evidence with item‐by‐item report is shown in Table 3.

**TABLE 3:** Grading of the evidence.

Variable	Consistency	Precision	Publication bias	Magnitude	Evidence
Age	↓	↓	=	↓	Very low ⊕◯◯◯
Years of education	↓	↓	↓	↓	Very low ⊕◯◯◯
Higher education	=	=	↓	↓	Low ⊕ ⊕ ◯◯
In a relationship	↓	↓	↓	↓	Very low ⊕◯◯◯
Age at onset	↓	↓	↓	↓	Very low ⊕◯◯◯
Psychotic features	=	=	↓	↓	Low ⊕ ⊕ ◯◯
History of suicide attempts	=	=	↓	↓	Low ⊕ ⊕ ◯◯
Number of previous manic episodes	↓	↓	↓	↓	Very low ⊕◯◯◯
Number of previous mixed episodes	=	=	↓	↓	Low ⊕ ⊕ ◯◯
Number of previous hospitalizations	=	↓	↓	=	Low ⊕ ⊕ ◯◯
Substance use disorder	↓	↓	=	=	Low ⊕ ⊕ ◯◯
Cannabis use disorder	=	=	↓	=	Moderate ⊕ ⊕ ⊕◯
Alcohol use disorder	=	=	↓	↓	Low ⊕ ⊕ ◯◯
Comorbid generalized anxiety disorder	=	↓	↓	=	Low ⊕ ⊕ ◯◯
Comorbid personality disorder	↓	↓	↓	↑	Low ⊕ ⊕ ◯◯
Global severity	↓	↓	↓	↓	Very low⊕◯◯◯
Insight	↓	↓	↓	=	Very low⊕◯◯◯
Global functioning	=	↓	↓	=	Low⊕ ⊕ ◯◯

## 4 Discussion

### 4.1 Summary and Interpretation of Findings

To our knowledge, this is the first systematic review and meta‐analysis specifically examining factors associated with poor medication adherence in people with BD. Identifying correlates of adherence could guide clinicians in the identification of those individuals who may most benefit from psychoeducational and behavioral interventions aimed at enhancing patients' awareness of the disorder [7, 51]. Based on data from 19 studies including a total of 38 variables of interest, our work provides valuable insights into factors associated with poor adherence in this clinical population, suggesting that adherence issues may vary across distinct subgroups of people with BD. Specifically, we found that individuals with poorer adherence tend to be younger, less educated, and less likely to be in a relationship. Also, they have an earlier age of onset and are more likely to show psychotic features, manic and mixed recurrences, with more hospital admissions, and a history of suicide attempts. In addition, poor adherence is associated with any substance use disorder (including alcohol and cannabis), as well as with other comorbid conditions, such as generalized anxiety and personality disorders, although for most of them resulting evidence was based on few studies and uncertain publication bias. Consistently, it is not surprising that these subjects also have higher global severity scores, less insight, and less global functioning than people adherent to medications. Interestingly, no gender differences were estimated in treatment adherence. This seems consistent with evidence that men and women with BD show no differences in factors known to impair adherence, such as self‐stigmatizing attitudes and perception of stigma [52]. This may also reflect possible differences in sampling procedures and gender distribution across studies.

However, it is important to interpret the findings of our work with caution, as the quality of evidence for most variables could be rated only as low or very low, with just cannabis use disorder based on moderate‐quality evidence, albeit derived from a limited number of studies. Issues such as small effects, imprecision, and inconsistency of overall estimates, together with the impossibility to rule out publication bias in most cases, must be considered.

Nonetheless, several important clinical considerations can be derived from these findings. A key point emerging from this meta‐analysis is that adherence in BD appears to be “time‐sensitive”. Specifically, younger patients tend to have poorer adherence. The association of poor adherence with earlier age at onset may be related to a combination of individual and clinical factors. Younger patients may be less likely to develop sufficient insight and awareness on the need for treatments. Moreover, this may also depend on specific clinical patterns of BD, as it is known that people with an earlier age at onset are more prone to develop a predominantly manic course [31], which often may impair adherence [53]. Our findings underscore the importance of delivering interventions designed to improve adherence since the first phases of the disorder. Early interventions may mitigate the negative outcomes associated with poor adherence, possibly reducing relapses and suicidal behaviors while enhancing psychosocial functioning. In particular, the relationship between poorer adherence and a history of suicide attempts from our findings is particularly noteworthy, considering that full adherence to lithium, the gold standard treatment for BD [54], might significantly reduce suicide risk [55]. Interestingly, poor medication adherence seems to correlate with a higher number of previous manic and mixed episodes but not of depressive ones.

A possible explanation is that individuals with BD may perceive depressive episodes as more distressful and problematic than manic ones [56, 57]. The subjective discomfort associated with depressive states may increase help‐seeking behavior and promote better adherence to treatment. In contrast, manic or mixed episodes are often experienced as less distressing due to elevated mood, heightened energy, and a temporary boost in psychosocial functioning [57, 58]: this relative lack of perceived impairment may contribute to poorer treatment engagement in individuals with a history of more mixed and manic episodes, as shown by our meta‐analysis. Further, recurrent mood episodes may reduce patients' confidence in the efficacy of mood stabilizers and other prescribed treatments for BD. It may thus be hypothesized that individuals with poorer adherence may also be those for whom maintenance treatment is less effective. This could also explain why patients with poor adherence seem more likely to misuse alcohol and illicit substances. Chronic substance use can induce neurobiological changes that may impair the clinical response to mood stabilizing drugs. Alternatively, individuals with poorer adherence may use alcohol and illicit substances to alleviate mood symptoms, particularly the depressive ones [59]. This can be true especially for cannabis use, that seems to be associated with poor adherence as well as manic relapses [60] and other negative outcomes, such as suicide [61]. Our findings suggest that personality disorders can also affect treatment adherence, since comorbid individuals are more prone to misusing alcohol and illicit substances [62]. Moreover, medications prescribed for BD do not have substantial effects on the dysfunctional personality traits potentially leading to low medication adherence [63]. However, our meta‐analysis was unable to differentiate between personality clusters, which likely contribute to treatment adherence in a heterogeneous manner. Moreover, there were not enough studies assessing affective temperaments, despite their potential influence on treatment adherence [64].

Overall, specific symptoms and co‐occurring conditions offer deeper understanding on reasons for poor adherence among individuals with BD. First, the presence of psychotic features in BD seems related to poor adherence to medications. Although non‐adherence is common across all psychiatric diagnoses, disorders characterized by psychotic symptoms pose additional challenges that further elevate the risk [65]. Psychotic features can directly impact the patient's insight, trust in medications, and ability to follow treatment recommendations. Moreover, our meta‐analysis reveals that individuals with generalized anxiety disorder are nearly four times more likely to exhibit poor adherence compared to those without. Comorbid anxiety disorders may worsen both the severity and clinical course of BD, potentially resulting in poorer outcomes and impaired functioning [66]. Indeed, anxiety may increase concerns about somatic symptoms, making these individuals more sensitive to medication side effects, which further leads to treatment discontinuation. It is essential to acknowledge that medication adherence can fluctuate with changes in the mental state of subjects with BD. Both psychotic features and anxiety, particularly when combined with affective symptoms, can significantly hinder treatment adherence [5, 65]. In addition, these clinical presentations often require complex treatments, which may further reduce patients' compliance.

While the relationship of adherence with both functioning and insight—some of the major correlates of therapeutic alliance and treatment outcomes in BD [67, 68]—might seem obvious, the lack of any association between negative attitudes and poor adherence is worth noting. This is surprising as negative attitudes have been generally considered to be closely related to adherence in BD [69]. However, this counterintuitive finding was mainly based on markedly inconsistent data on patients' attitude towards medications from three studies which used heterogeneous assessment methods.

Another interesting finding emerging from our meta‐analysis is that people with poorer adherence are less likely to be married. This is consistent with evidence emerging from a seminal meta‐analysis highlighting that, along with other social factors, being married, especially in a context of cohesive families, might improve adherence to prescribed treatments [70]. Our findings may indirectly support the hypothesis that psychoeducation might play a key role in improving outcomes of patients with BD, enhancing their attitude towards medications, and favoring their compliance to treatments [9, 71]. Finally, being less educated seems to correlate with poorer adherence, possibly because lower educational attainments may affect the understanding of the disorder, the need for treatment, and the characteristics of prescribed medications, overall impacting adherence [72, 73].

However, because of the low‐to‐very low evidence emerging from our meta‐analysis, additional studies are needed to explore possible factors influencing the relationship between these variables and medication adherence in BD. In addition, research should also target potential factors associated with poor adherence to non‐pharmacological treatments, since different psychotherapeutic interventions, such as psychoeducation and cognitive‐behavioral treatments, are key components of BD management [74]. Moreover, as this meta‐analysis explores clinical and psychosocial correlates of poor adherence, further studies are needed to explore additional neurobiological correlates. For instance, network‐based alterations in task‐induced functional connectivity recently described in BD [75] may represent determinants of poor adherence worthy of future investigation.

## 5 Limitations

The findings of the current systematic review and meta‐analysis should be interpreted with caution considering some limitations. First, since our work investigated cross‐sectional differences between poor and good adherence, we cannot draw any conclusions about the possible causal relationships between the tested variable and outcomes. Second, although we included only studies using clearly defined and reliable assessments for adherence in order to limit the risk of inconsistency and misclassification, we estimated a high between‐study heterogeneity (I^2^ > 80%) for several analyses. While this degree of heterogeneity is not unexpected in behavioral research, it may be due to several factors, including assessment methods (e.g., clinical rating scales vs. objective biological measures), variability in population characteristics (e.g., illness severity and treatment regimens), and geographical or cultural differences across study settings, which may influence both adherence behaviors and their clinical determinants. Moreover, it is worth mentioning that there is no gold standard approach for measuring adherence in individuals with BD [76] and mental disorders in general [65]. As highlighted in other research based on a similar approach [31, 77], other sources of bias such as poor sample representativeness and recall bias (due to the nature of some of tested variables), may have affected the results of our meta‐analysis. Third, due to the observational design of the included studies, we should consider that, at least for some variables, the likelihood of selective reporting bias may have somehow influenced the meta‐analytic estimates. In addition, although our meta‐analysis uncovered several clinical and sociodemographic correlates, there were no sufficient data to test other important variables that may have a potential effect on adherence in people with BD, such as the occurrence of side effects [9], the predominant polarity [78, 79], and affective temperaments [64].

## 6 Conclusions

This systematic review and meta‐analysis provides a comprehensive overview of the factors associated with poor medication adherence in individuals with BD, highlighting key sociodemographic and clinical characteristics of this population that may guide targeted early interventions in high‐risk groups. Future research should also explore the causal pathways linking clinical factors to poor adherence and examine additional determinants to help refine adherence‐enhancing strategies in BD.

## Supporting information


**Figure S1:** Distribution of included studies by year of publication.
**Figure S2:** Mean age of participants with poor adherence as compared with those with good adherence.
**Figure S3:** Male gender in participants with poor adherence as compared with those with good adherence.
**Figure S4:** Mean years of education of participants with poor adherence as compared with those with good adherence.
**Figure S5:** Higher education in participants with poor adherence as compared with those with good adherence.
**Figure S6:** Being in a relationship in participants with poor adherence as compared with those with good adherence.
**Figure S7:** Unemployment in participants with poor adherence as compared with those with good adherence.
**Figure S8:** Living alone in participants with poor adherence as compared with those with good adherence.
**Figure S9:** Age at onset in participants with poor adherence as compared with those with good adherence.
**Figure S10:** Duration of illness in participants with poor adherence as compared with those with good adherence.
**Figure S11:** Psychotic features in participants with poor adherence as compared with those with good adherence.
**Figure S12:** History of suicide attempts in participants with poor adherence as compared with those with good adherence.
**Figure S13:** Family history of mood disorders in participants with poor adherence as compared with those with good adherence.
**Figure S14:** Diagnosis of bipolar disorder type I in participants with poor adherence as compared with those with good adherence.
**Figure S15:** rapid cycling course in participants with poor adherence as compared with those with good adherence.
**Figure S16:** Number of previous manic episodes in participants with poor adherence as compared with those with good adherence.
**Figure S17:** Number of previous mixed episodes in participants with poor adherence as compared with those with good adherence.
**Figure S18:** Total number of previous mood episodes in participants with poor adherence as compared with those with good adherence.
**Figure S19:** Number of previous depressive episodes in participants with poor adherence as compared with those with good adherence.
**Figure S20:** Number of previous hospitalizations in participants with poor adherence as compared with those with good adherence.
**Figure S21:** Having had at least one previous hospitalization in participants with poor adherence as compared with those with good adherence.
**Figure S22:** Substance use disorder in participants with poor adherence as compared with those with good adherence.
**Figure S23:** Cannabis use disorder in participants with poor adherence as compared with those with good adherence.
**Figure S24:** Alcohol use disorder in participants with poor adherence as compared with those with good adherence.
**Figure S25:** Comorbid generalized anxiety disorder in participants with poor adherence as compared with those with good adherence.
**Figure S26:** Comorbid personality disorder in participants with poor adherence as compared with those with good adherence.
**Figure S27:** Polypharmacotherapy in participants with poor adherence as compared with those with good adherence.
**Figure S28:** Lithium use in participants with poor adherence as compared with those with good adherence.
**Figure S29:** Valproate use in participants with poor adherence as compared with those with good adherence.
**Figure S30:** Carbamazepine use in participants with poor adherence as compared with those with good adherence.
**Figure S31:** Antidepressant use in participants with poor adherence as compared with those with good adherence.
**Figure S32:** Antipsychotic use in participants with poor adherence as compared with those with good adherence.
**Figure S33:** First‐generation antipsychotic use in participants with poor adherence as compared with those with good adherence.
**Figure S34:** Second‐generation antipsychotic use in participants with poor adherence as compared with those with good adherence.
**Figure S35:** Global severity scores in participants with poor adherence as compared with those with good adherence.
**Figure S36:** Insight scores in participants with poor adherence as compared with those with good adherence.
**Figure S37:** Global functioning scores in participants with poor adherence as compared with those with good adherence.
**Figure S38:** Presence of general medical comorbidities in participants with poor adherence as compared with those with good adherence.
**Figure S39:** Drug attitude scores in participants with poor adherence as compared with those with good adherence.

## Data Availability

The data that support the findings of this study are available from the corresponding author upon reasonable request.
