# Supplementary material for: Clinical and Sociodemographic Correlates of Poor Medication Adherence in People With Bipolar Disorder: A Systematic Review and Meta‐Analysis
Source: Bipolar Disord. 2026 Jun 10;28(5):e70127. doi: 10.1111/bdi.70127 (PMC13251348; doi:10.1111/bdi.70127)
Supplement: Supplementary file 1 — Figure S1: Distribution of included studies by year of publication. Figure S2: Mean age of participants with poor adherence as compared with those with good adherence. Figure S3: Male gender in participants with poor adherence as compared with those with good adherence. Figure S4: Mean years of education of participants with poor adherence as compared with those with good adherence. Figure S5: Higher education in participants with poor adherence as compared with those with good adherence. Figure S6: Being in a relationship in participants with poor adherence as compared with those with good adherence. Figure S7: Unemployment in participants with poor adherence as compared with those with good adherence. Figure S8: Living alone in participants with poor adherence as compared with those with good adherence. Figure S9: Age at onset in participants with poor adherence as compared with those with good adherence. Figure S10: Duration of illness in participants with poor adherence as compared with those with good adherence. Figure S11: Psychotic features in participants with poor adherence as compared with those with good adherence. Figure S12: History of suicide attempts in participants with poor adherence as compared with those with good adherence. Figure S13: Family history of mood disorders in participants with poor adherence as compared with those with good adherence. Figure S14: Diagnosis of bipolar disorder type I in participants with poor adherence as compared with those with good adherence. Figure S15: rapid cycling course in participants with poor adherence as compared with those with good adherence. Figure S16: Number of previous manic episodes in participants with poor adherence as compared with those with good adherence. Figure S17: Number of previous mixed episodes in participants with poor adherence as compared with those with good adherence. Figure S18: Total number of previous mood episodes in participants with poor adherence as compared with those with go [file BDI-28-0-s001.docx]

**Supporting information**

**Clinical and sociodemographic correlates of poor medication adherence in people with bipolar disorder: A systematic review and meta-analysis**

Francesco Bartoli^1,^*, Daniele Cavaleri^1^, Ilaria Riboldi^1^, Chiara Alessandra Capogrosso^1^, Carlo Bassetti^1^, Marco Broccia^1^, Giorgio Cucchi^1^, Cristina Crocamo^1^, Martha Sajatovic^2^, Giuseppe Carrà^1^.

^1^ School of Medicine and Surgery, University of Milano-Bicocca, via Cadore 48, 20900 Monza, Italy.

^2^ Neurological and Behavioral Outcomes Center, University Hospitals Cleveland Medical Center, Case Western Reserve University, Cleveland, OH, 44106, USA.

Supplementary figures: 39

Abbreviations: k = number of studies; N = total number of participants; OR = odds ratio; SMD = standardized mean difference; CI = confidence interval; I² = heterogeneity statistic.

**Supplementary** **Figure 1.** Distribution of included studies by year of publication.


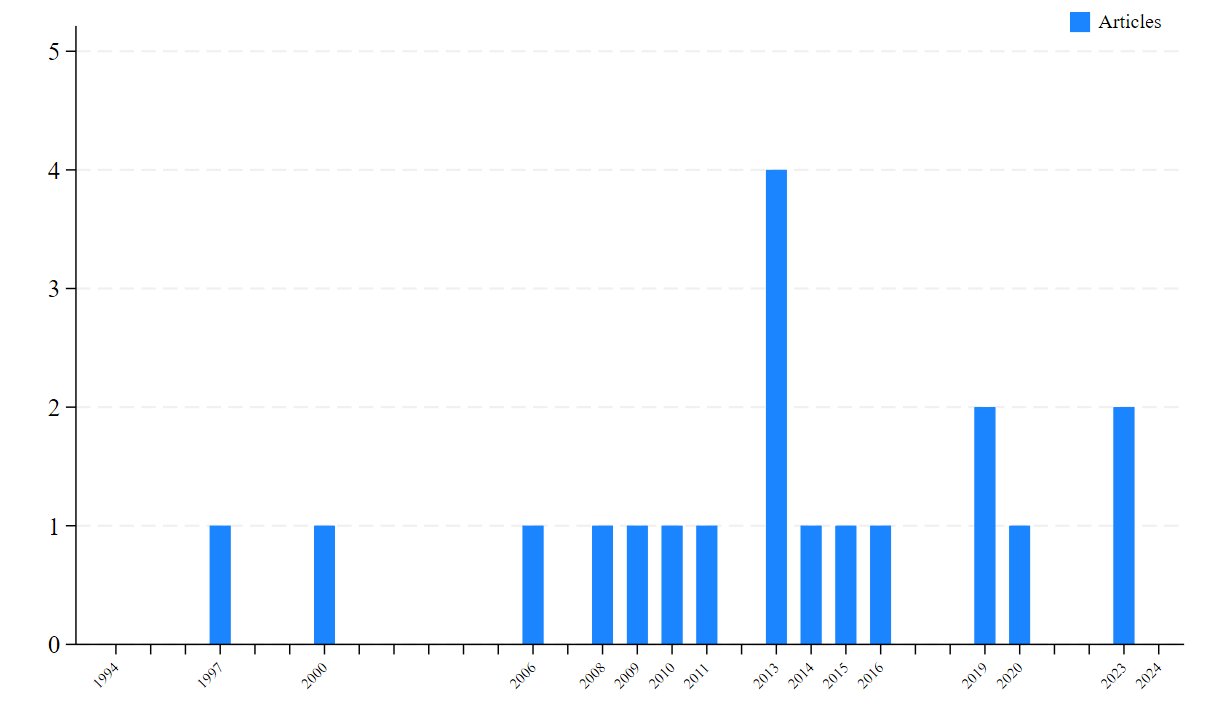


**Supplementary** **Figure 2.** Mean age of participants with poor adherence as compared with those with good adherence.


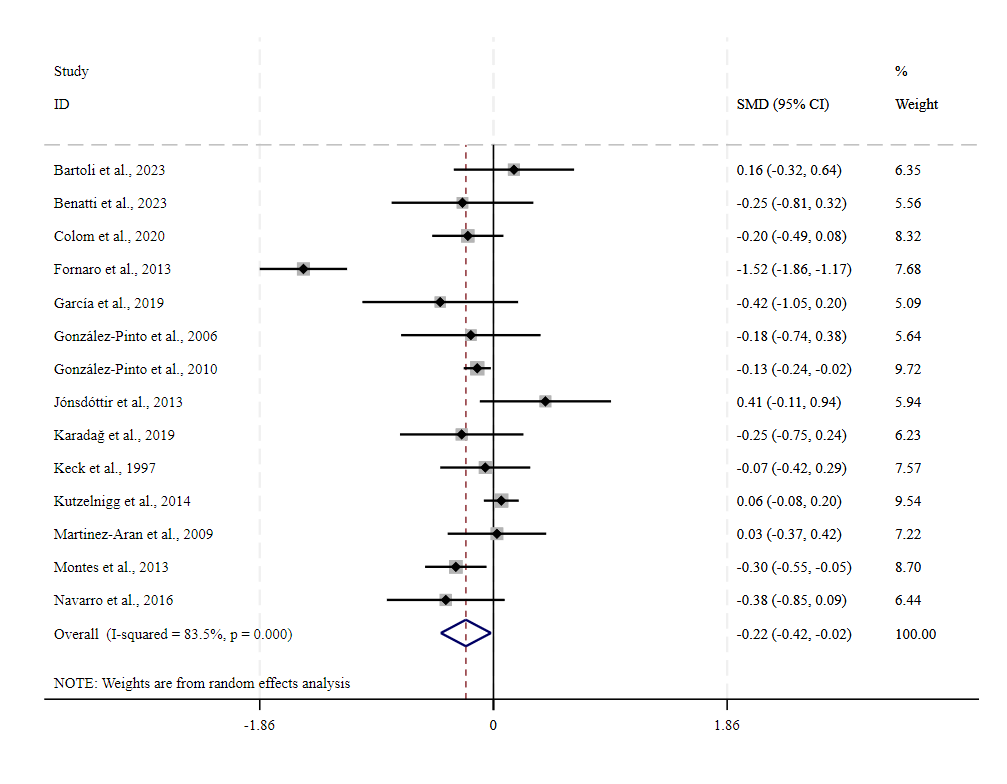


k=14; N=4,220; SMD=–0.22, 95%CI: –0.42 to –0.02, p=0.031; I²=83.5%

**Supplementary** **Figure 3.** Male gender in participants with poor adherence as compared with those with good adherence.


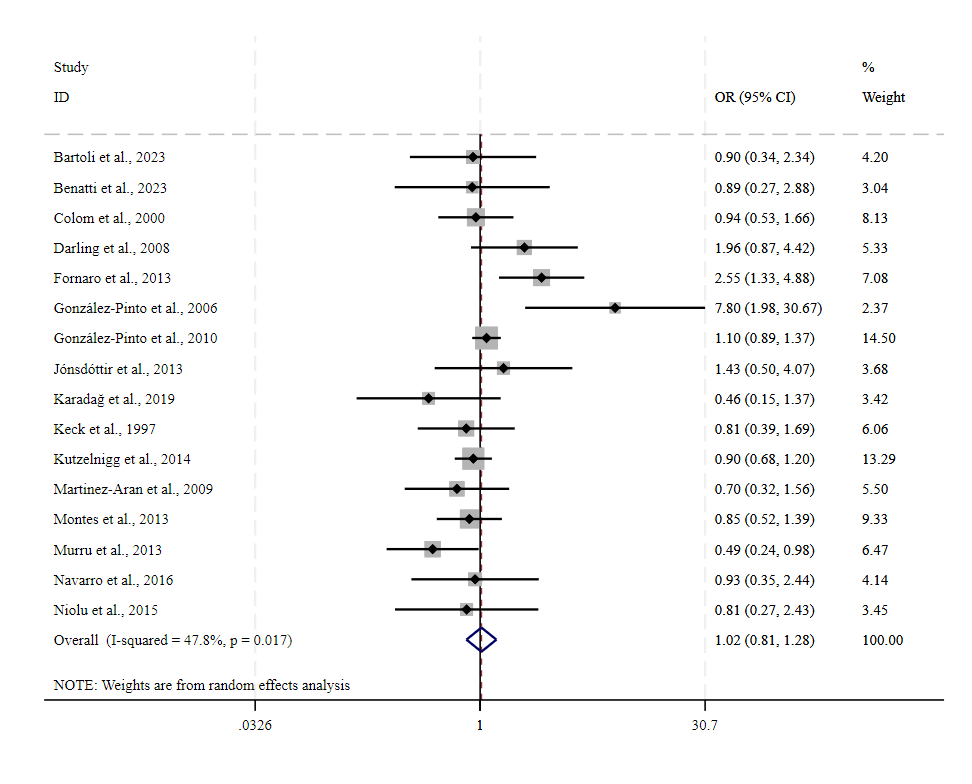


k=16; N=4,517; OR=1.02, 95%CI: 0.81 to 1.28, p=0.867; I²=47.8%

**Supplementary** **Figure 4.** Mean years of education of participants with poor adherence as compared with those with good adherence.


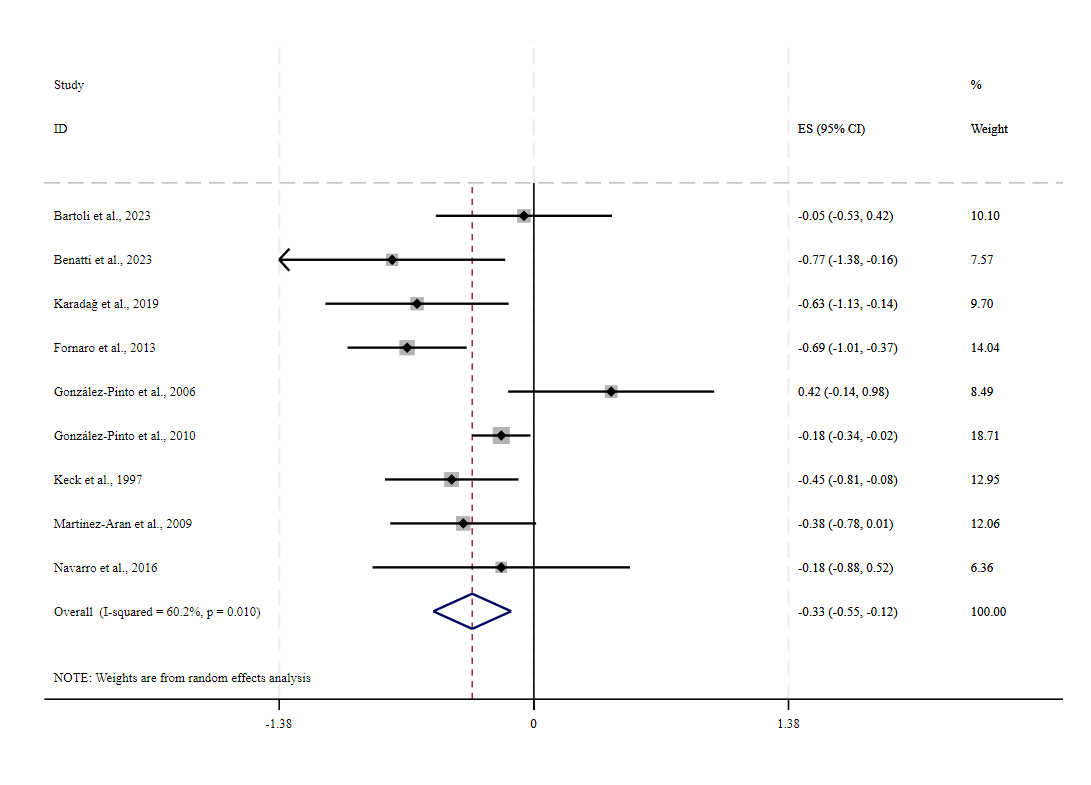


k=9; N=2,675; SMD=–0.34, 95%CI: –0.55 to –0.12, p=0.002; I²=60.2%

**Supplementary** **Figure 5.** Higher education in participants with poor adherence as compared with those with good adherence.


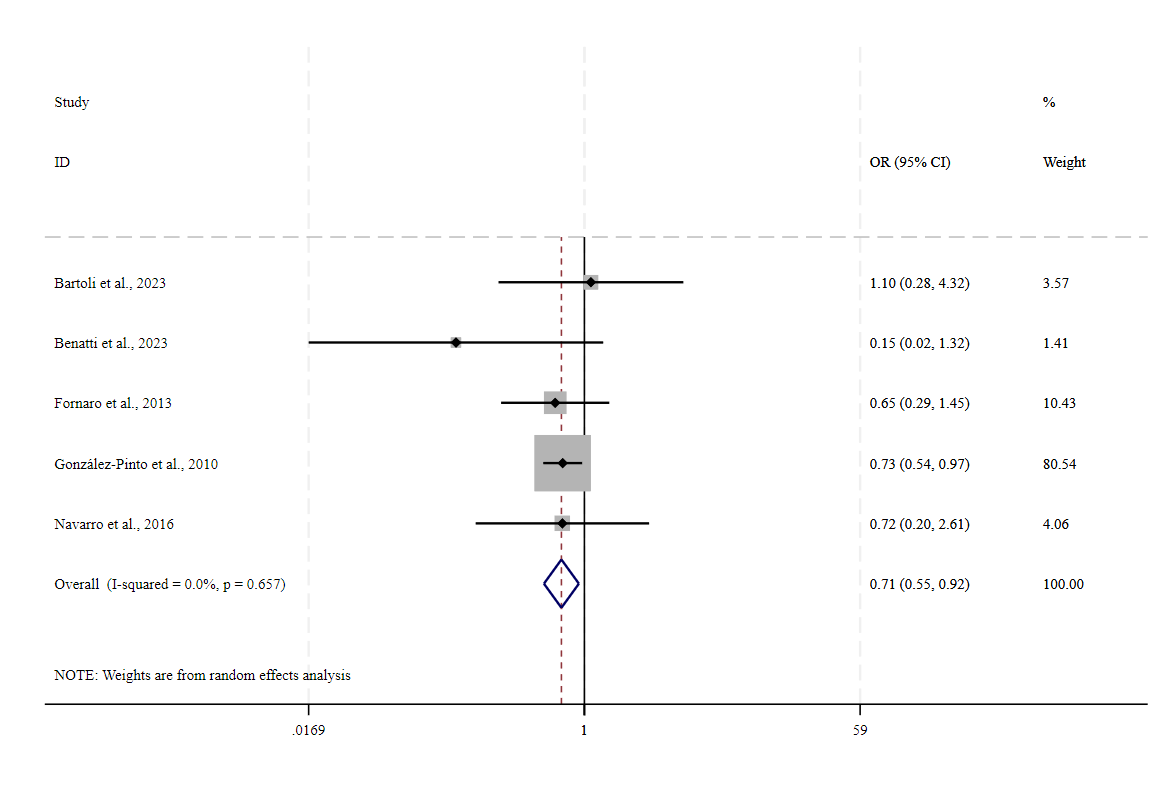


k=5; N=2,249; OR=0.71, 95%CI: 0.55 to 0.92, p=0.010; I²=0%

**Supplementary** **Figure 6.** Being in a relationship in participants with poor adherence as compared with those with good adherence.


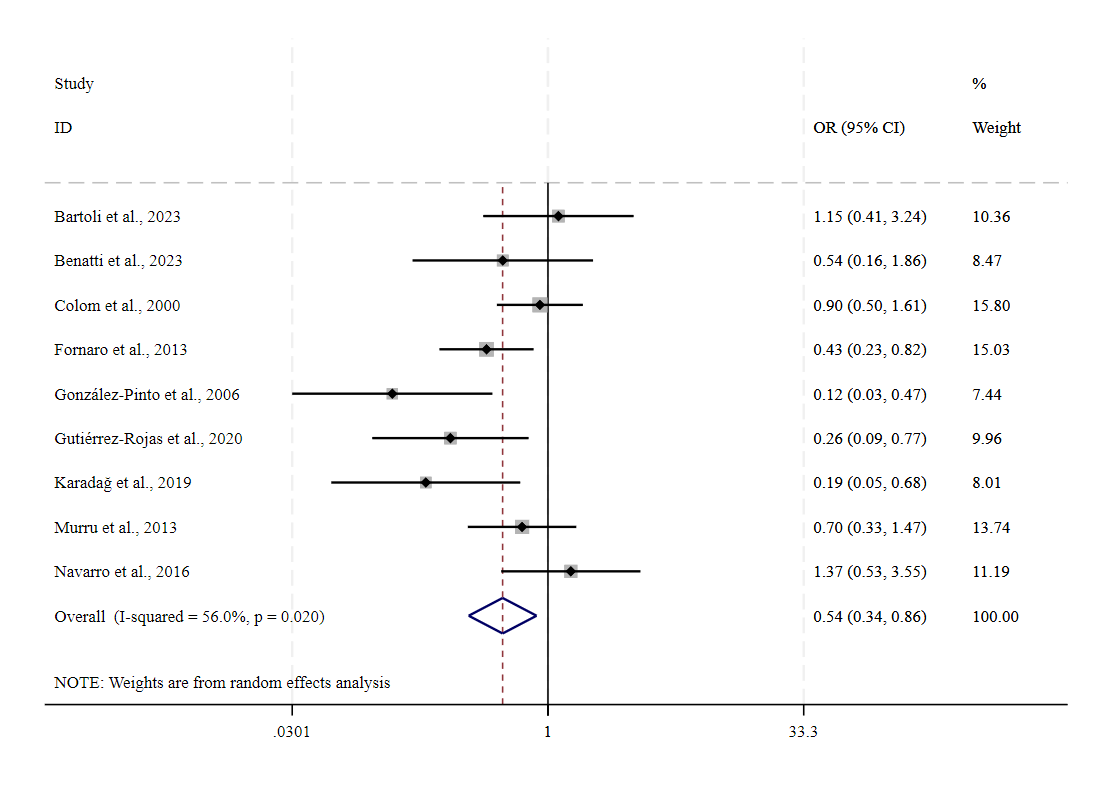


k=9; N=1,056; OR=0.54, 95%CI: 0.34 to 0.86, p=0.009; I²=56.0%

**Supplementary** **Figure 7.** Unemployment in participants with poor adherence as compared with those with good adherence.


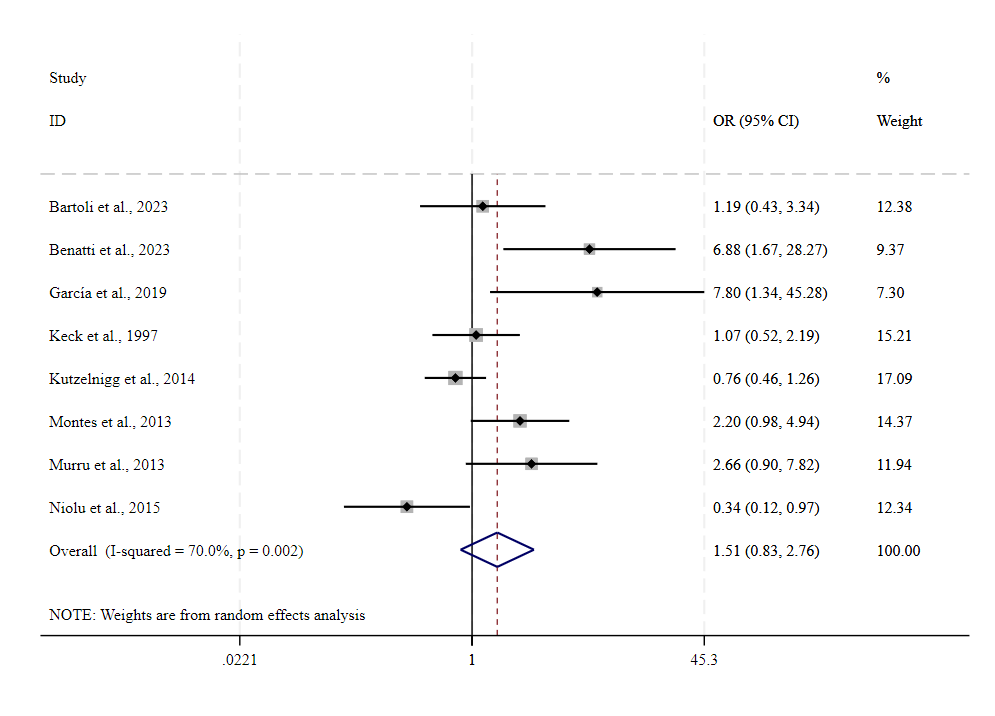


k=8; N=1,730; OR=1.51, 95%CI: 0.83 to 2.76, p=0.177; I²=70.0%

**Supplementary** **Figure 8.** Living alone in participants with poor adherence as compared with those with good adherence.


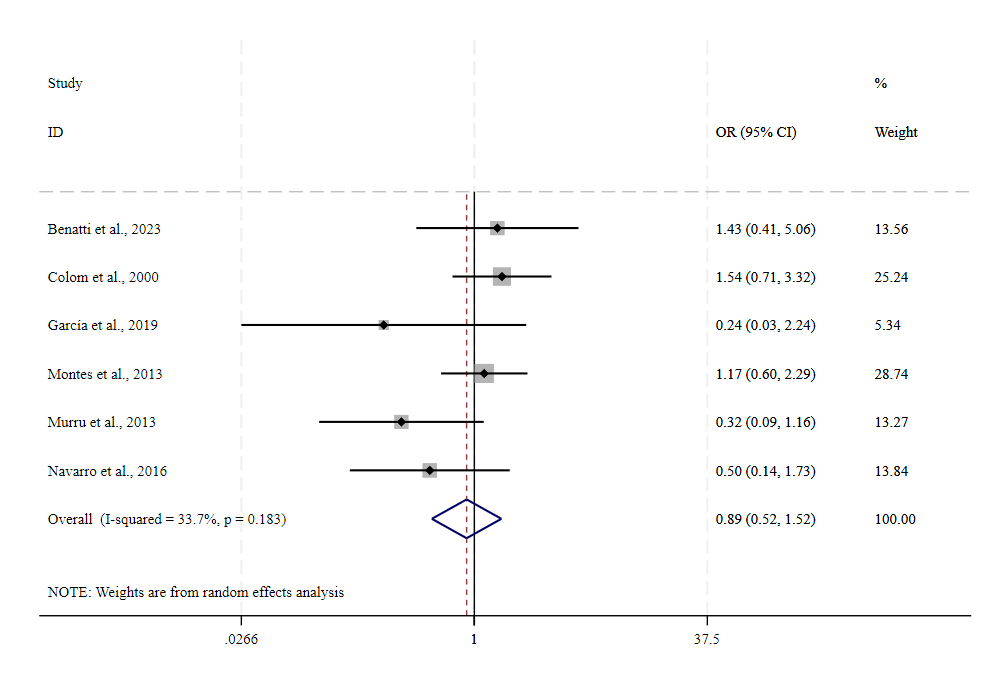


k=6; N=814; OR=0.89, 95%CI: 0.52 to 1.52, p=0.664; I²=33.7%

**Supplementary** **Figure 9.** Age at onset in participants with poor adherence as compared with those with good adherence.


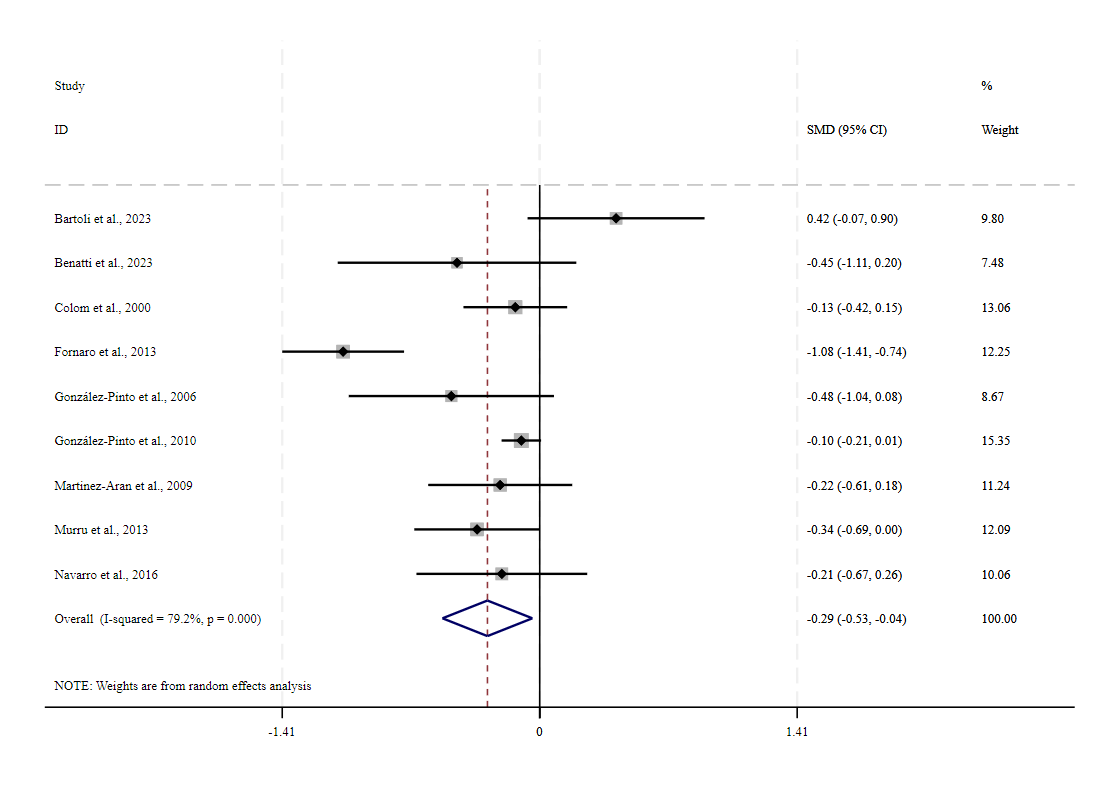


k=9; N=2,761; SMD=–0.29, 95%CI: –0.53 to –0.04, p=0.023; I²=79.2%

**Supplementary** **Figure 10.** Duration of illness in participants with poor adherence as compared with those with good adherence.


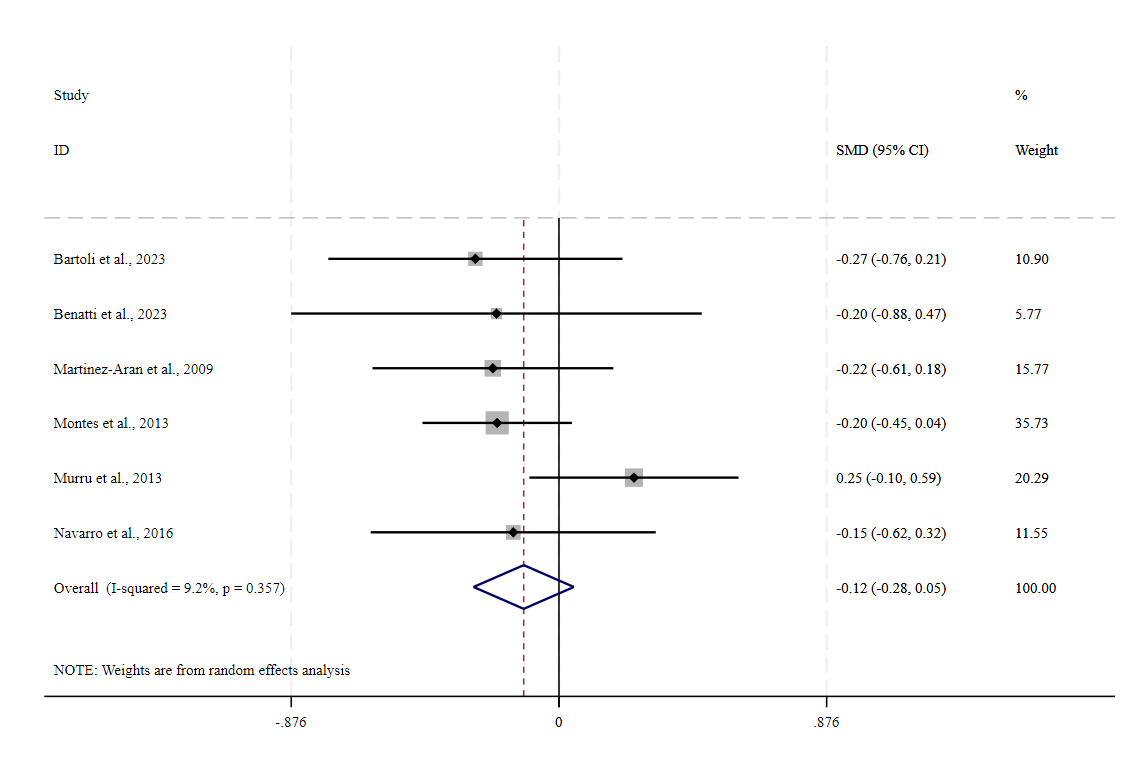


k=6; N=739; SMD=–0.12, 95%CI: –0.28 to 0.05, p=0.168; I²=9.2%

**Supplementary** **Figure 11.** Psychotic features in participants with poor adherence as compared with those with good adherence.


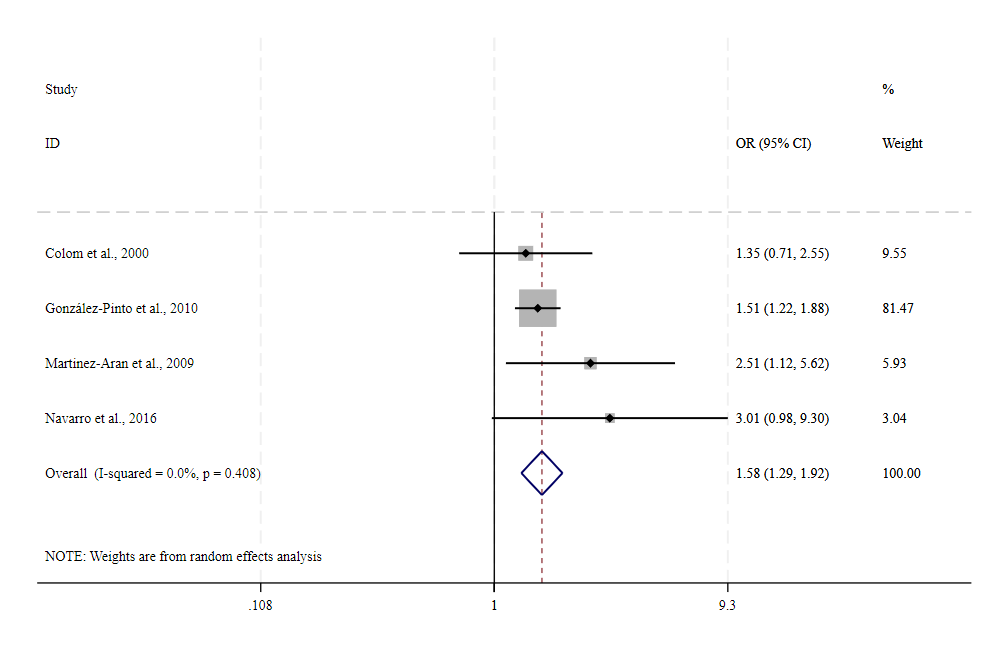


k=4; N=2,210; OR=1.58, 95%CI: 1.30 to 1.92, p<0.001; I²=0%

**Supplementary** **Figure 12.** History of suicide attempts in participants with poor adherence as compared with those with good adherence.


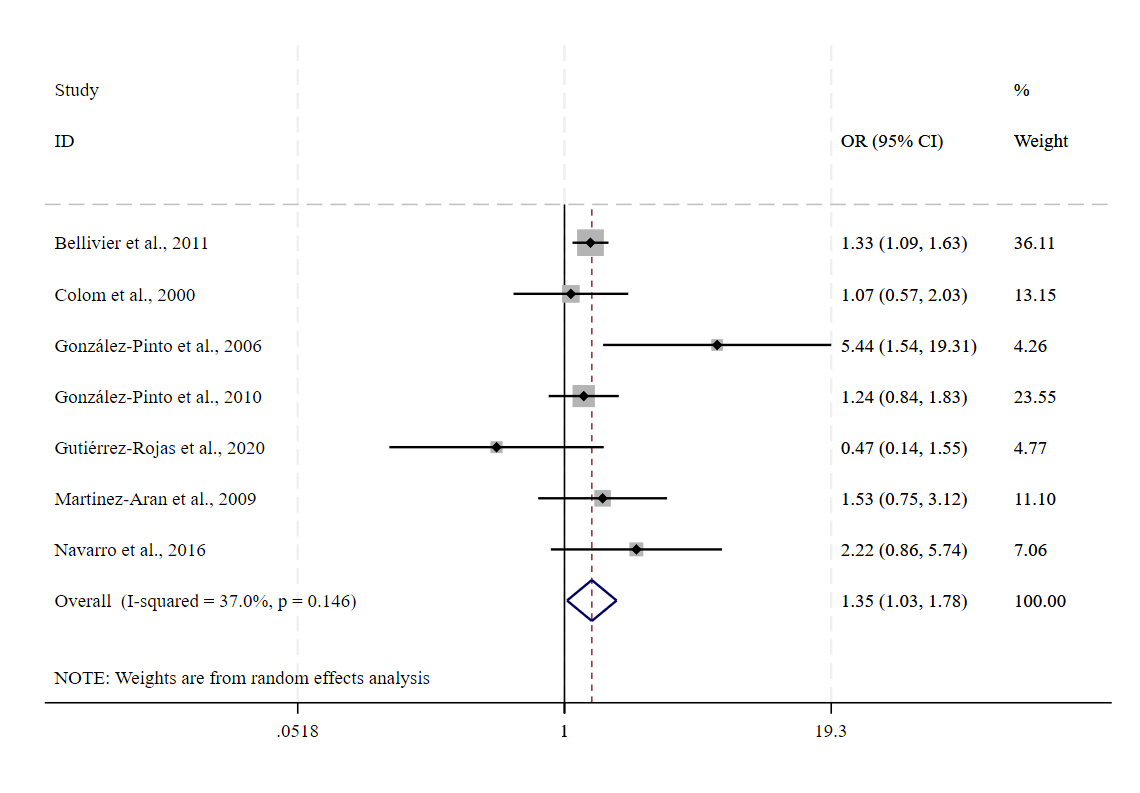


k=7; N=4,609; OR=1.36, 95%CI: 1.03 to 1.78, p=0.030; I²=37.0%

**Supplementary** **Figure 13.** Family history of mood disorders in participants with poor adherence as compared with those with good adherence.


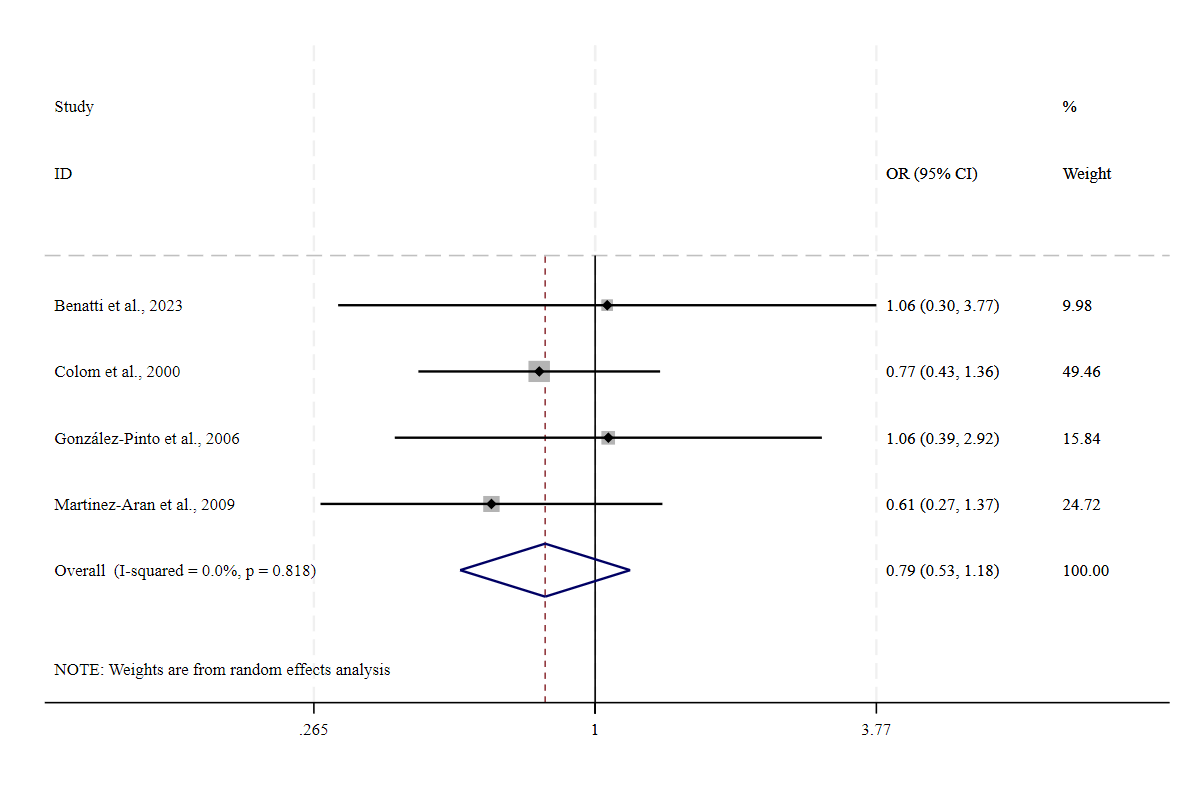


k=4; N=419; OR=0.79, 95%CI: 0.53 to 1.18, p=0.249; I²=0%

**Supplementary** **Figure 14.** Diagnosis of bipolar disorder type I in participants with poor adherence as compared with those with good adherence.


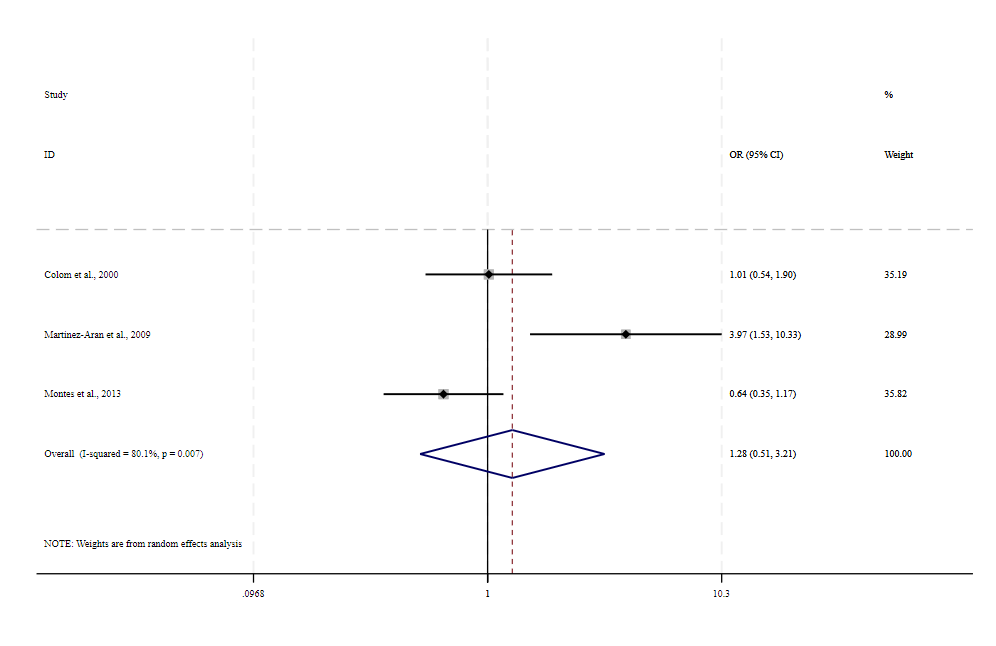


k=3; N=606; OR=1.28, 95%CI: 0.51 to 3.21, p=0.599; I²=80.1%

**Supplementary** **Figure 15.** Rapid cycling course in participants with poor adherence as compared with those with good adherence.


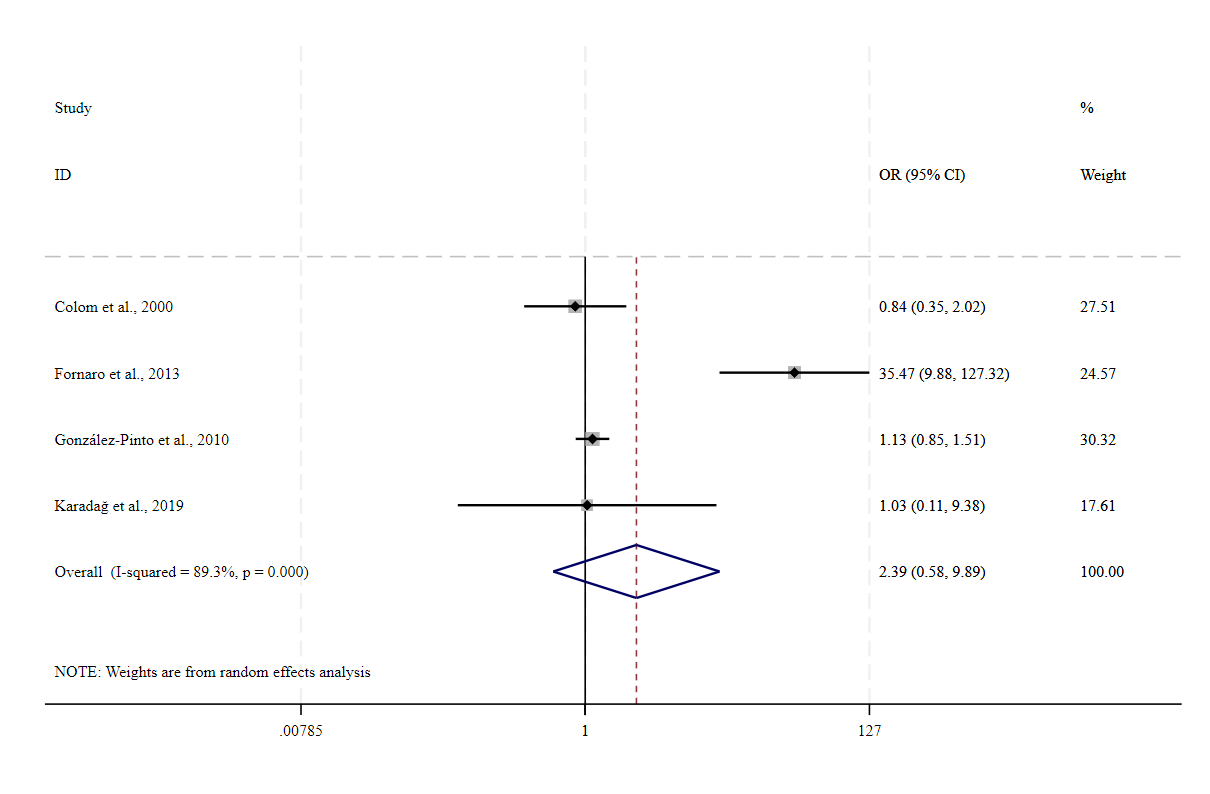


k=4; N=2,368; OR=2.40, 95%CI: 0.58 to 9.89, p=0.705; I²=89.3%

**Supplementary** **Figure 16.** Number of previous manic episodes in participants with poor adherence as compared with those with good adherence.


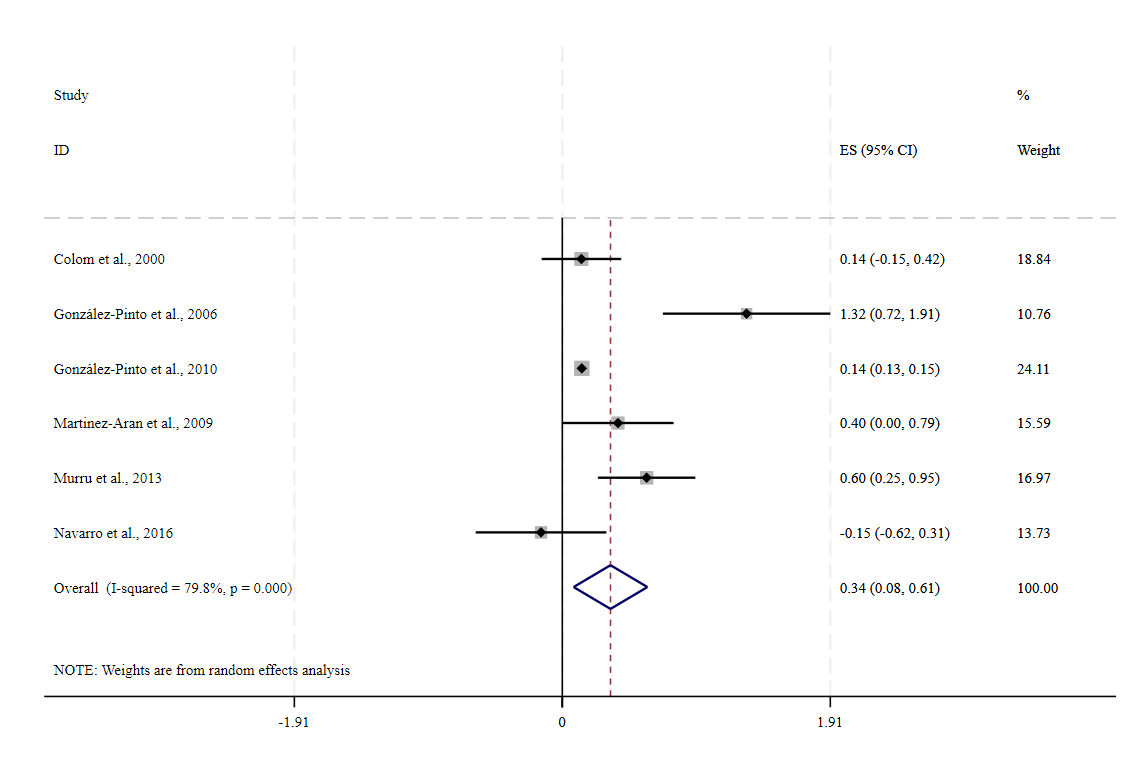


k=6; N=2,432; SMD=0.34, 95%CI: 0.08 to 0.61, p=0.011; I²=79.8%

**Supplementary** **Figure 17.** Number of previous mixed episodes in participants with poor adherence as compared with those with good adherence.


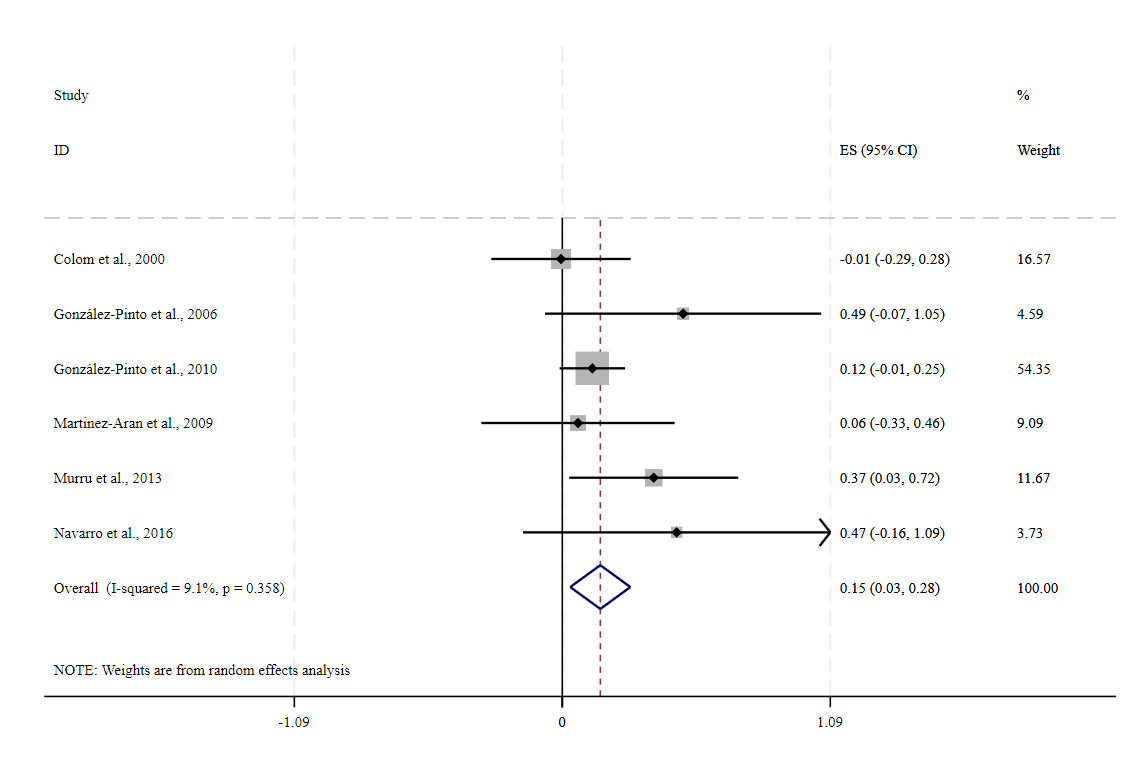


k=6; N=2,432; SMD=0.16, 95%CI: 0.03 to 0.28, p=0.013; I²=9.1%

**Supplementary** **Figure 18.** Total number of previous mood episodes in participants with poor adherence as compared with those with good adherence.


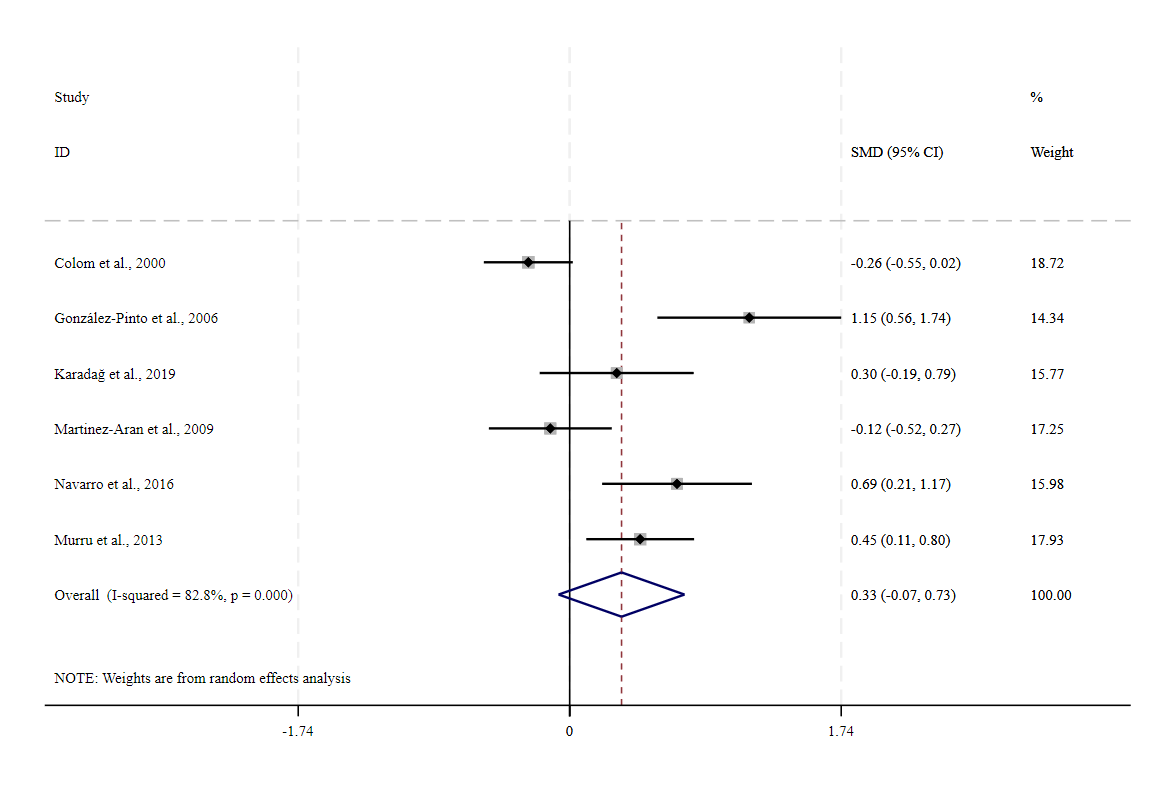


k=6; N=718; SMD=0.33, 95%CI: –0.07 to 0.73, p=0.106; I²=82.8%

**Supplementary** **Figure 19.** Number of previous depressive episodes in participants with poor adherence as compared with those with good adherence.


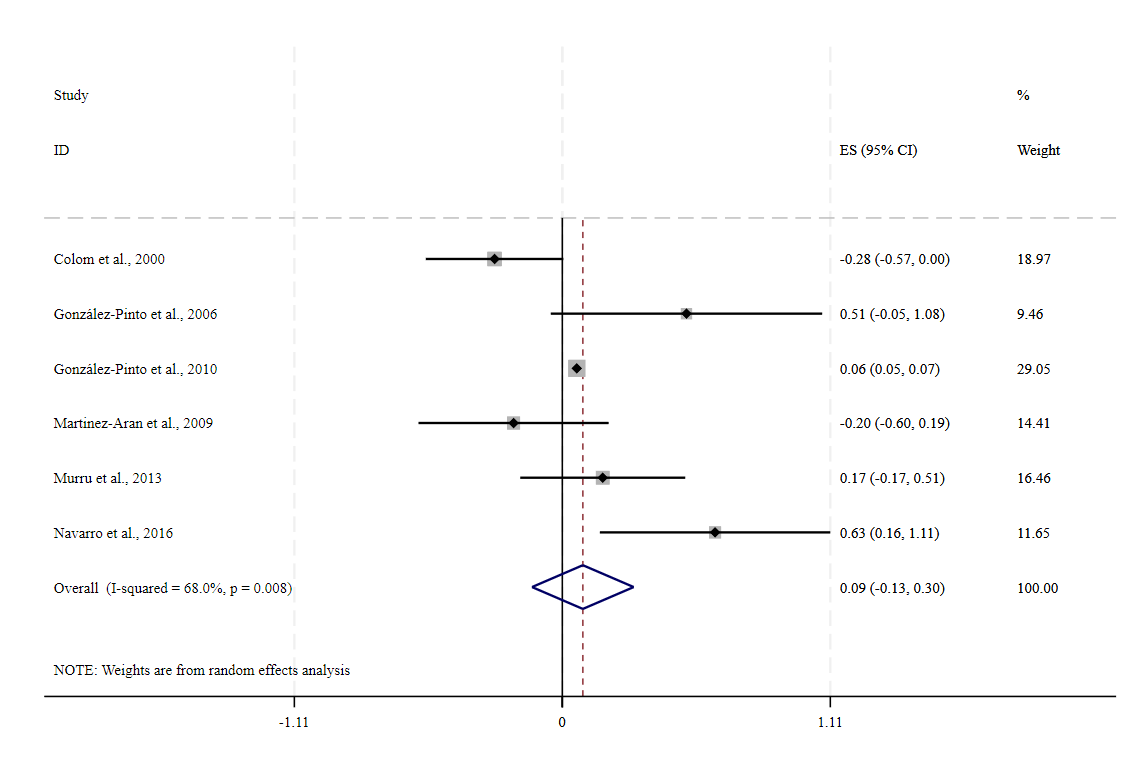


k=6; N=2,432; SMD=0.08, 95%CI: –0.13 to 0.30, p=0.429; I²=68.0%

**Supplementary** **Figure 20.** Number of previous hospitalizations in participants with poor adherence as compared with those with good adherence.


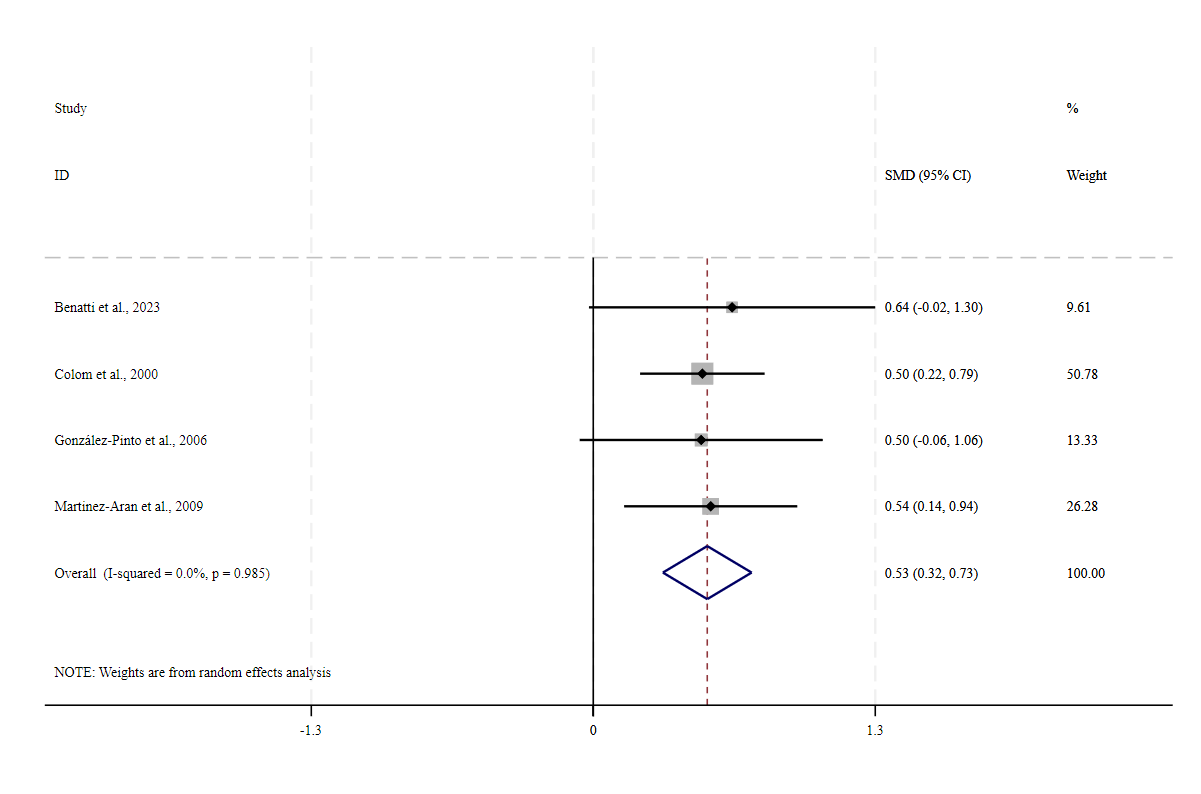


k=4; N=413; SMD=0.53, 95%CI: 0.32 to 0.73, p<0.001; I²=0%

**Supplementary** **Figure 21.** Having had at least one previous hospitalization in participants with poor adherence as compared with those with good adherence.


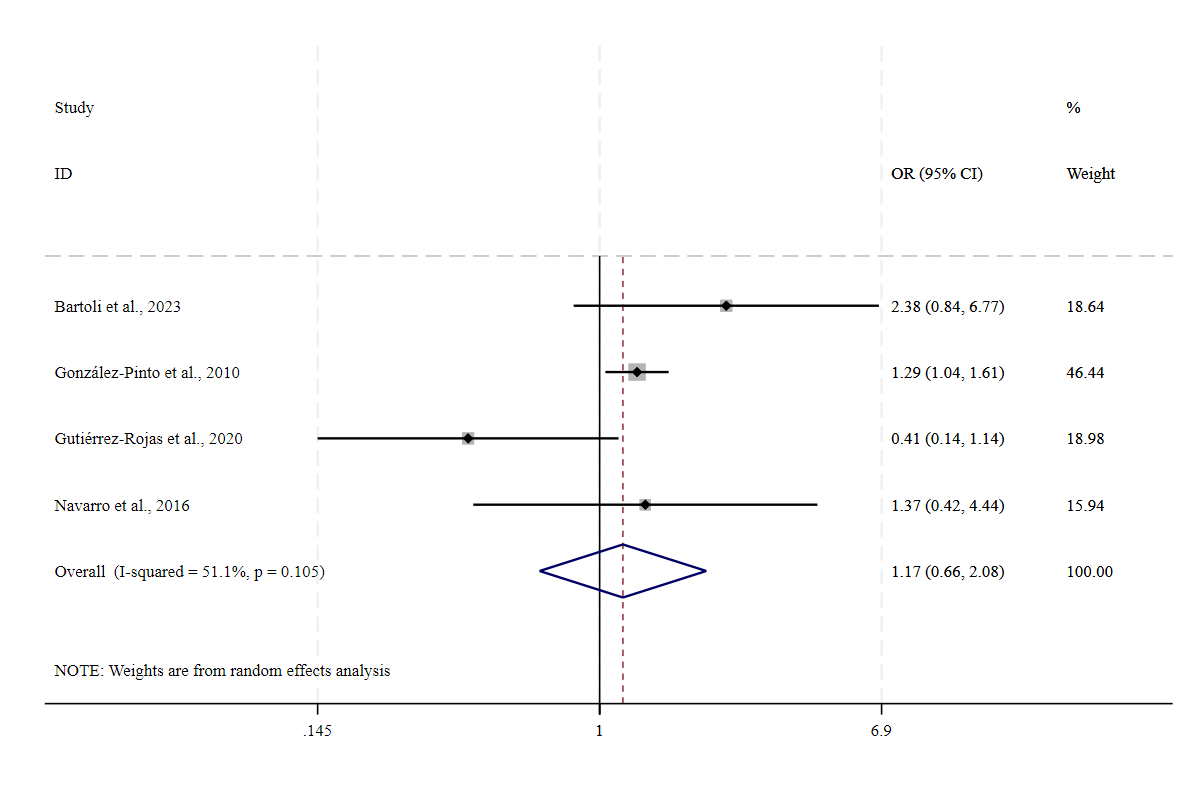


k=4; N=2,086; OR=1.17, 95%CI: 0.66 to 2.08, p=0.583; I²=51.1%

**Supplementary** **Figure 22.** Substance use disorder in participants with poor adherence as compared with those with good adherence.


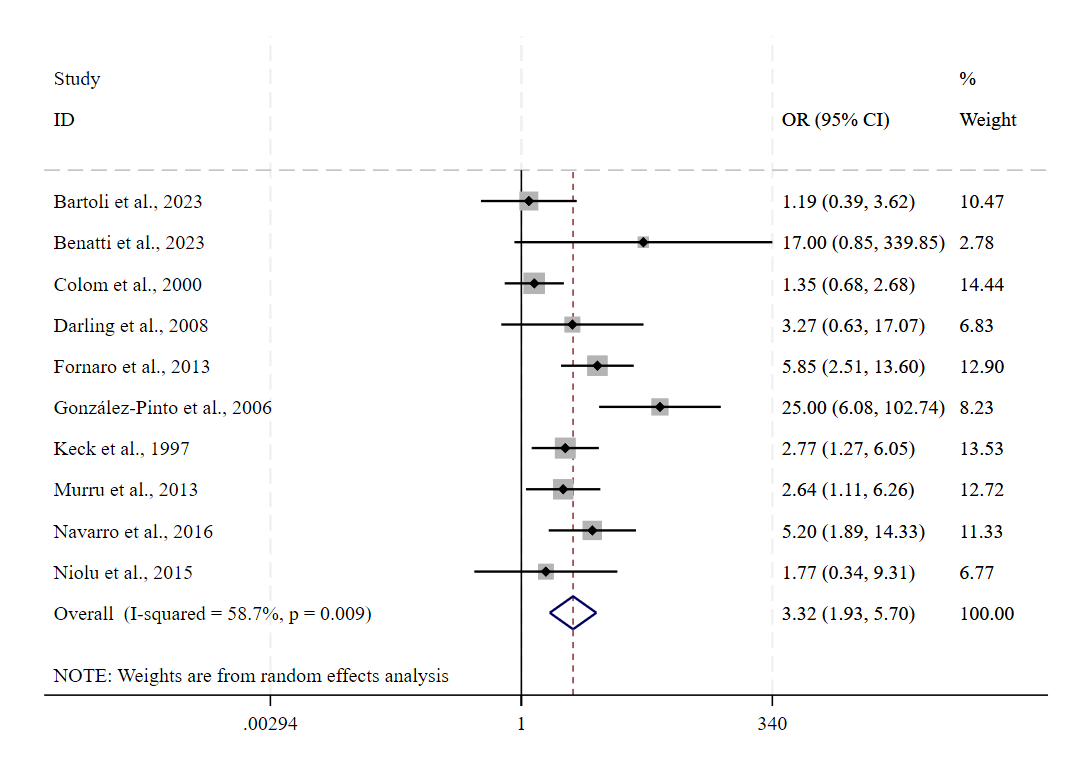


k=10; N=1,159; OR=3.32, 95%CI: 1.94 to 5.70, p<0.001; I²=58.7%

**Supplementary** **Figure 23.** Cannabis use disorder in participants with poor adherence as compared with those with good adherence.


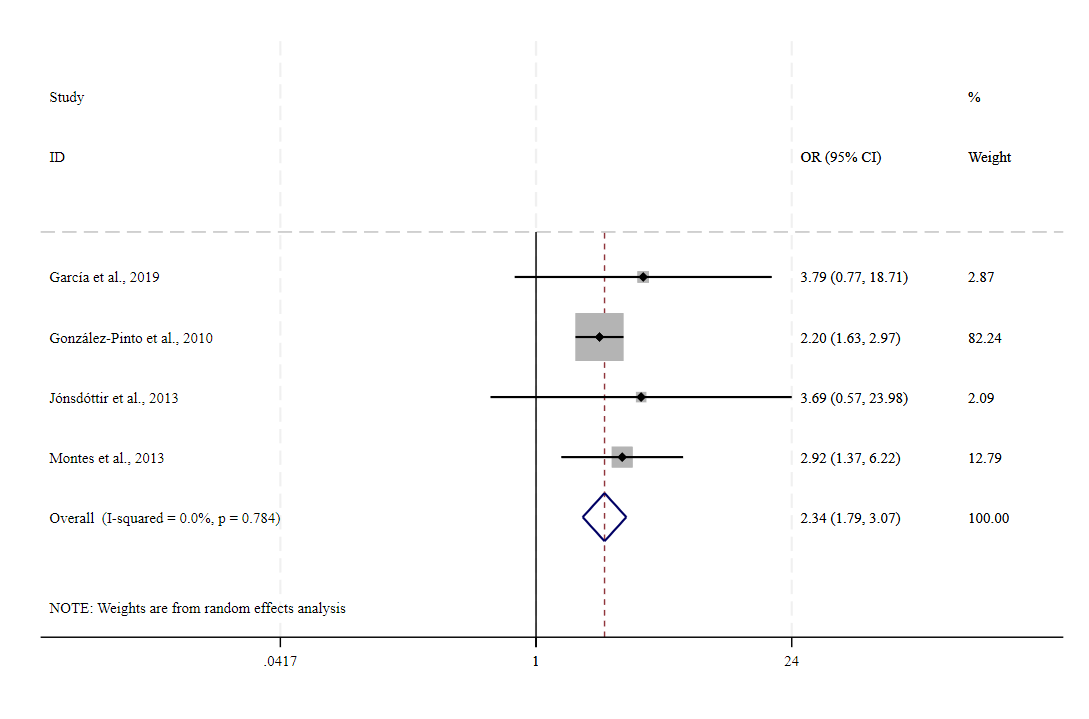


k=4; N=2,281; OR=2.34, 95%CI: 1.79 to 3.07, p<0.001; I²=0%

**Supplementary** **Figure 24.** Alcohol use disorder in participants with poor adherence as compared with those with good adherence.


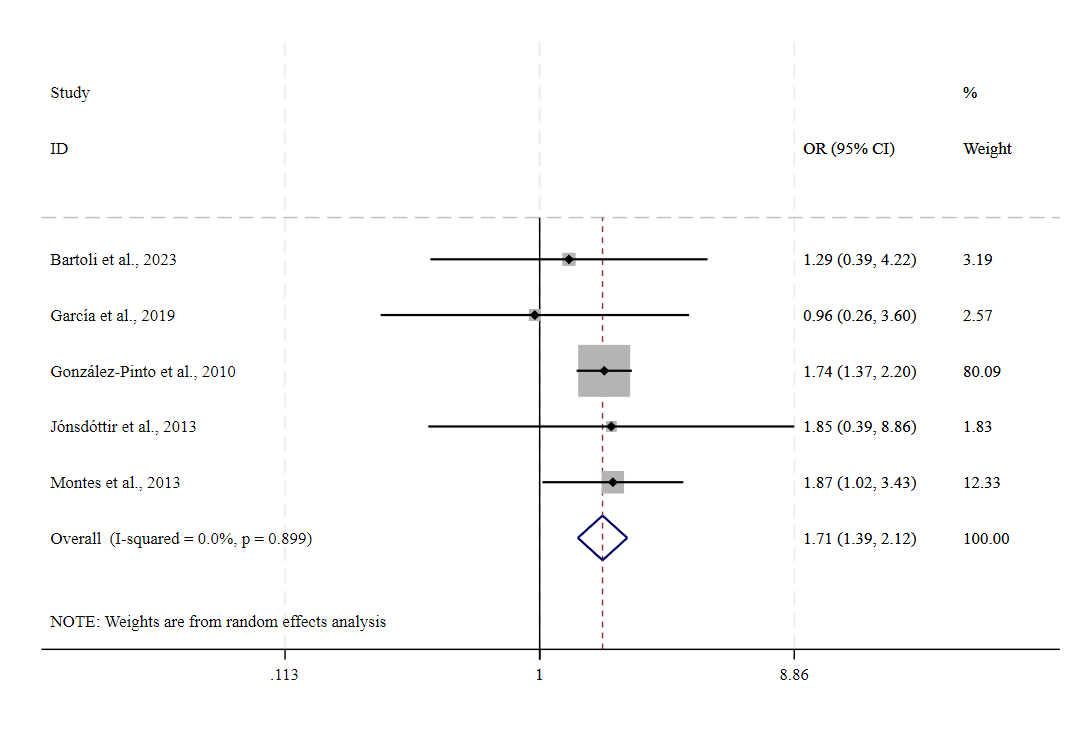


k=5; N=2,352; OR=1.71, 95%CI: 1.39 to 2.12, p<0.001; I²=0%

**Supplementary** **Figure 25.** Comorbid generalized anxiety disorder in participants with poor adherence as compared with those with good adherence.


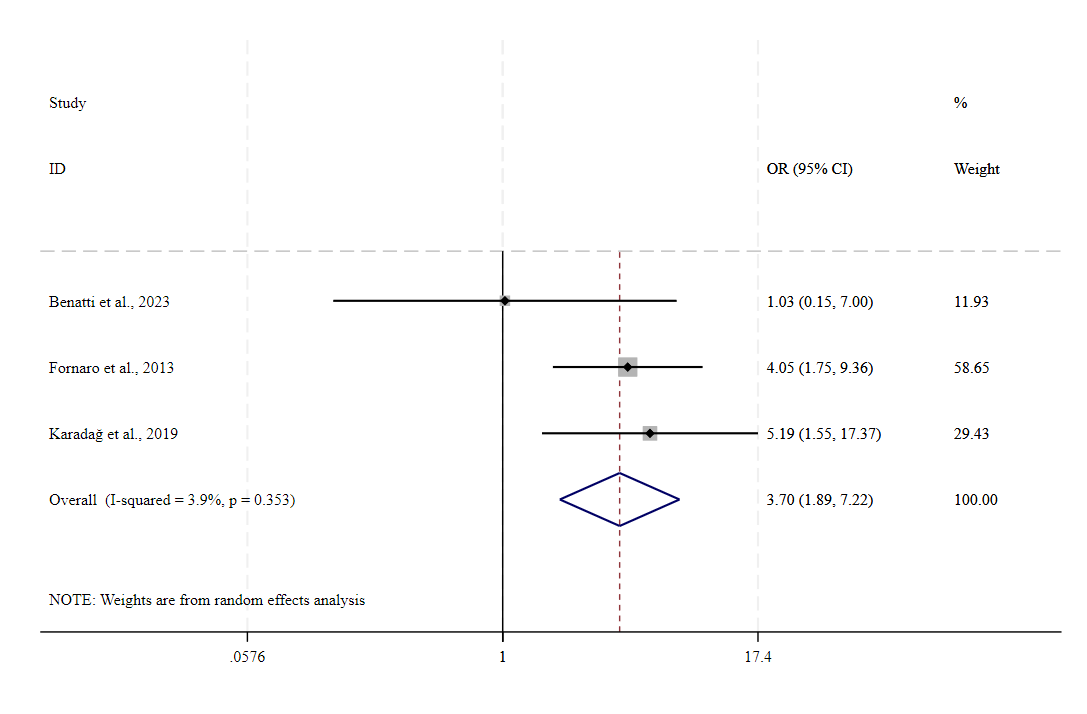


k=3; N=375; OR=3.70, 95%CI: 1.90 to 7.22, p<0.001; I²=3.9%

**Supplementary** **Figure 26.** Comorbid personality disorder in participants with poor adherence as compared with those with good adherence.


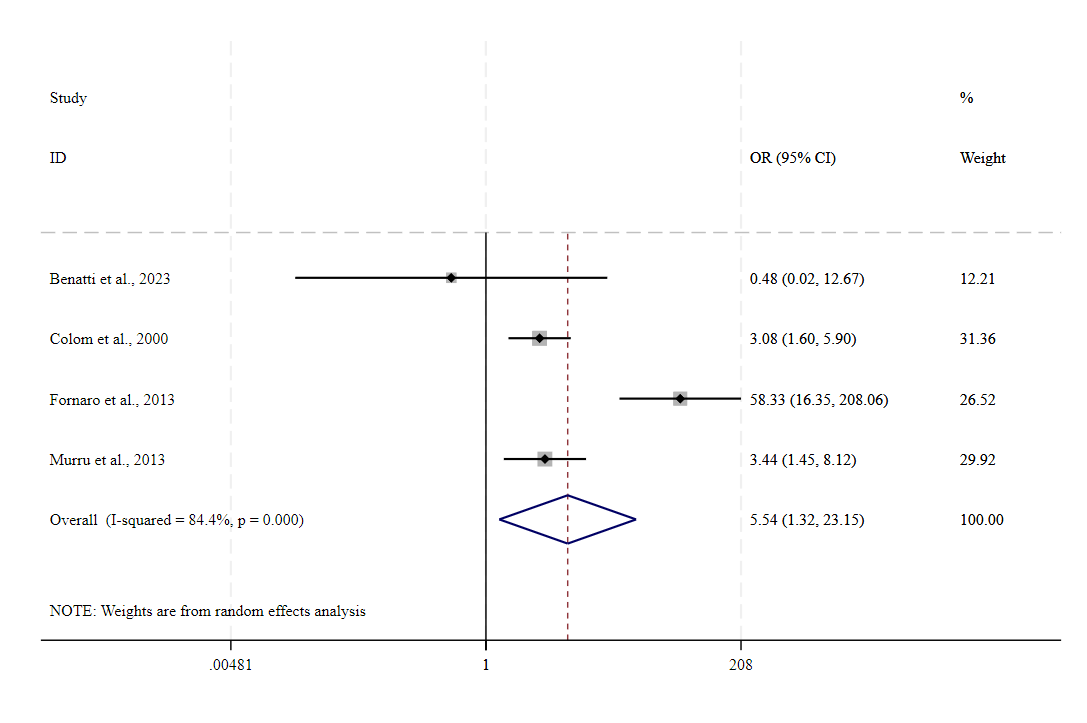


k=4; N=605; OR=5.54, 95%CI: 1.32 to 23.15, p=0.019; I²=84.4%

**Supplementary** **Figure 27.** Polypharmacotherapy in participants with poor adherence as compared with those with good adherence.


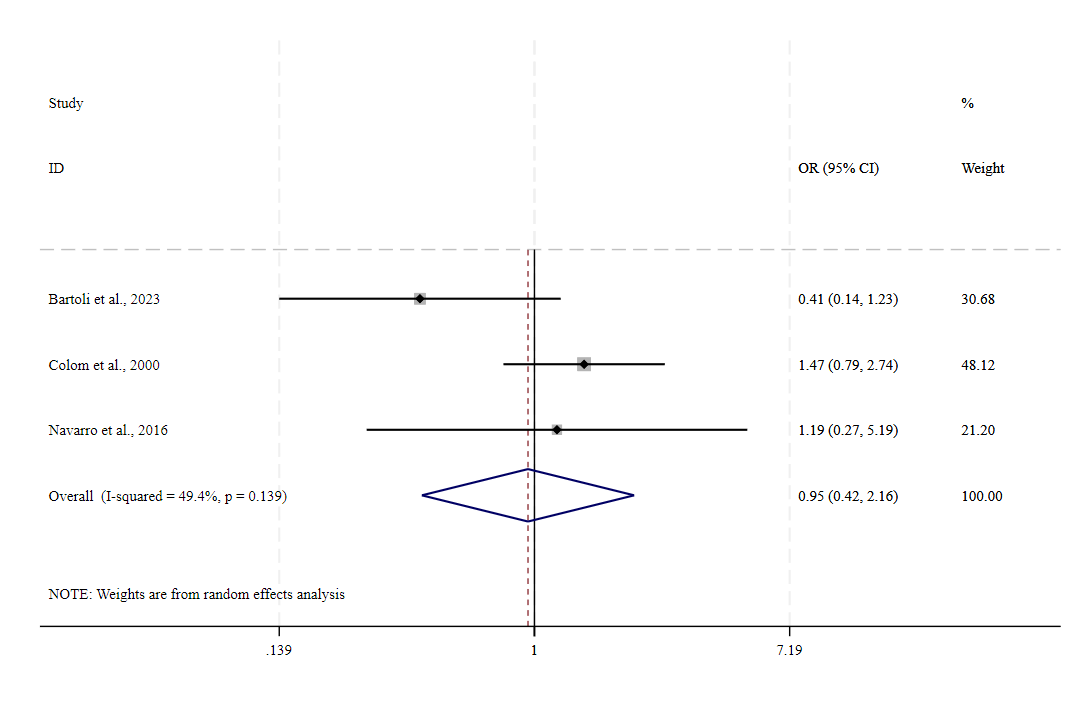


k=3; N=347; OR=0.95, 95%CI: 0.42 to 2.16, p=0.905; I²=49.4%

**Supplementary** **Figure 28.** Lithium use in participants with poor adherence as compared with those with good adherence.


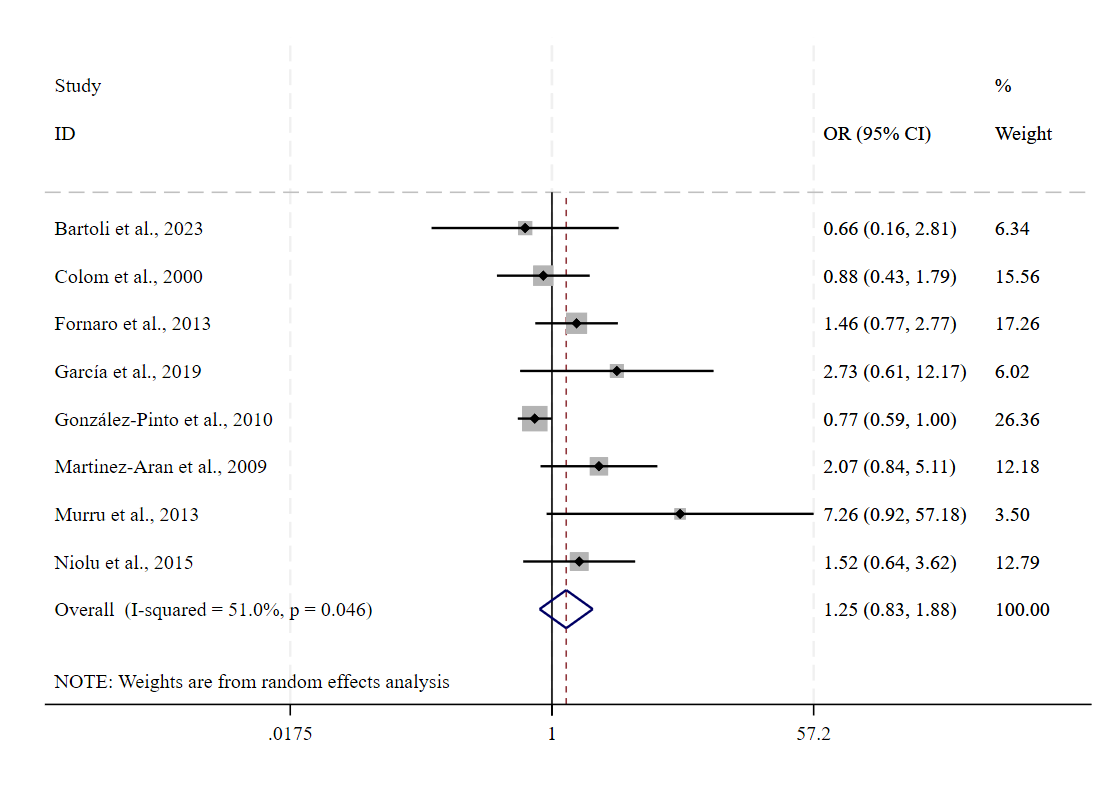


k=8; N=2,706; OR=1.25, 95%CI: 0.83 to 1.88, p=0.293; I²=51.0%

**Supplementary** **Figure 29.** Valproate use in participants with poor adherence as compared with those with good adherence.


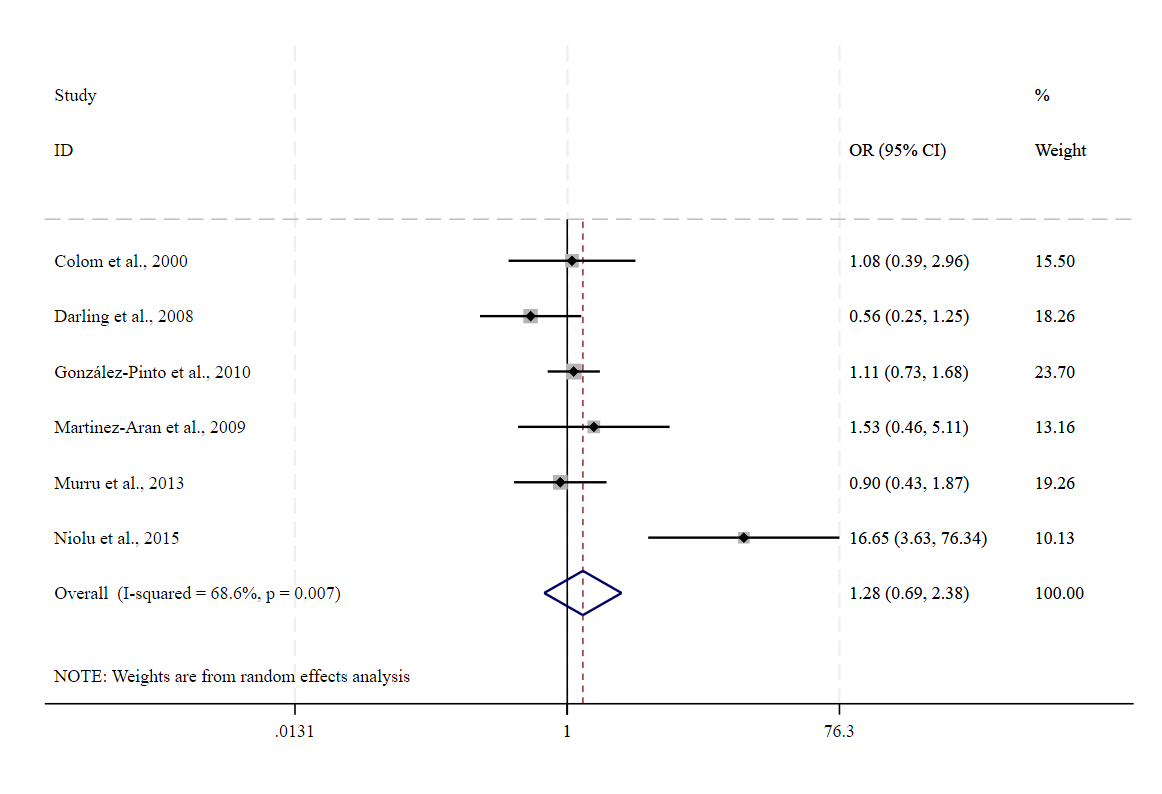


k=6; N=2,463; OR=1.28, 95%CI: 0.69 to 2.38, p=0.428; I²=68.6%

**Supplementary** **Figure 30.** Carbamazepine use in participants with poor adherence as compared with those with good adherence.


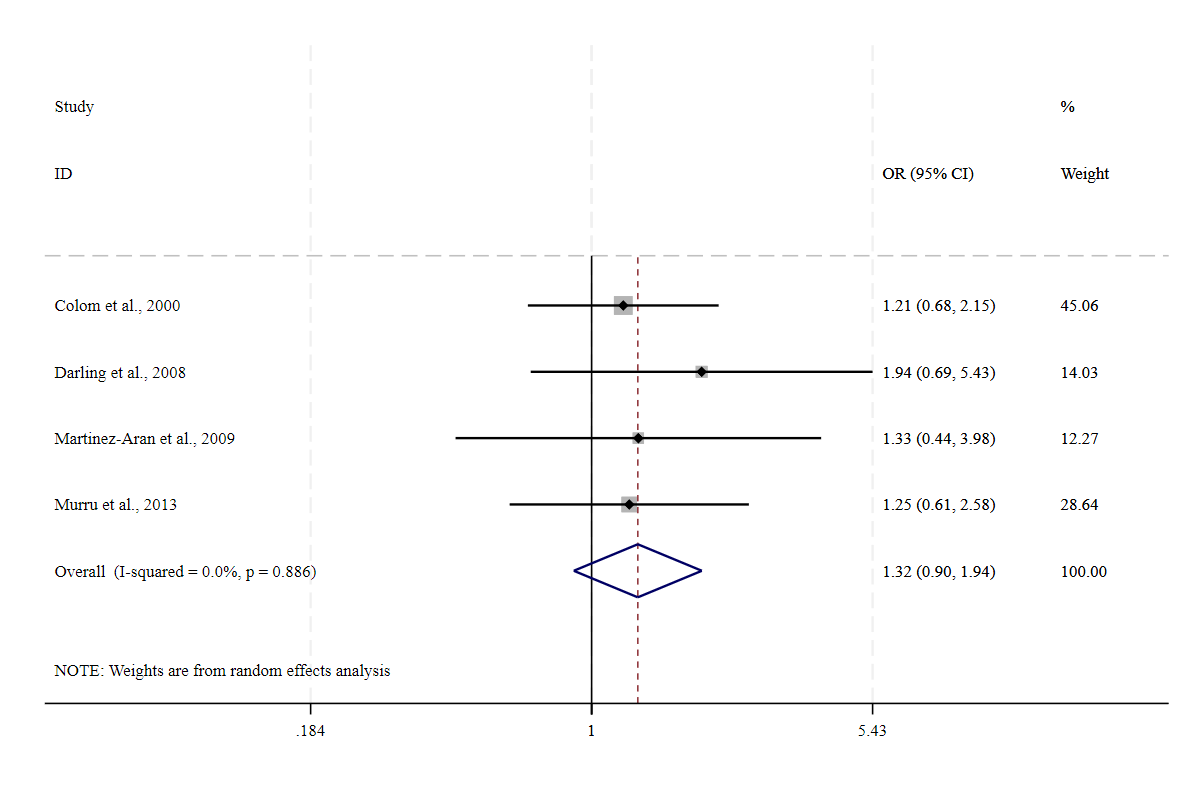


k=4; N=544; OR=1.32, 95%CI: 0.90 to 1.94, p=0.157; I²=0%

**Supplementary** **Figure 31.** Antidepressant use in participants with poor adherence as compared with those with good adherence.


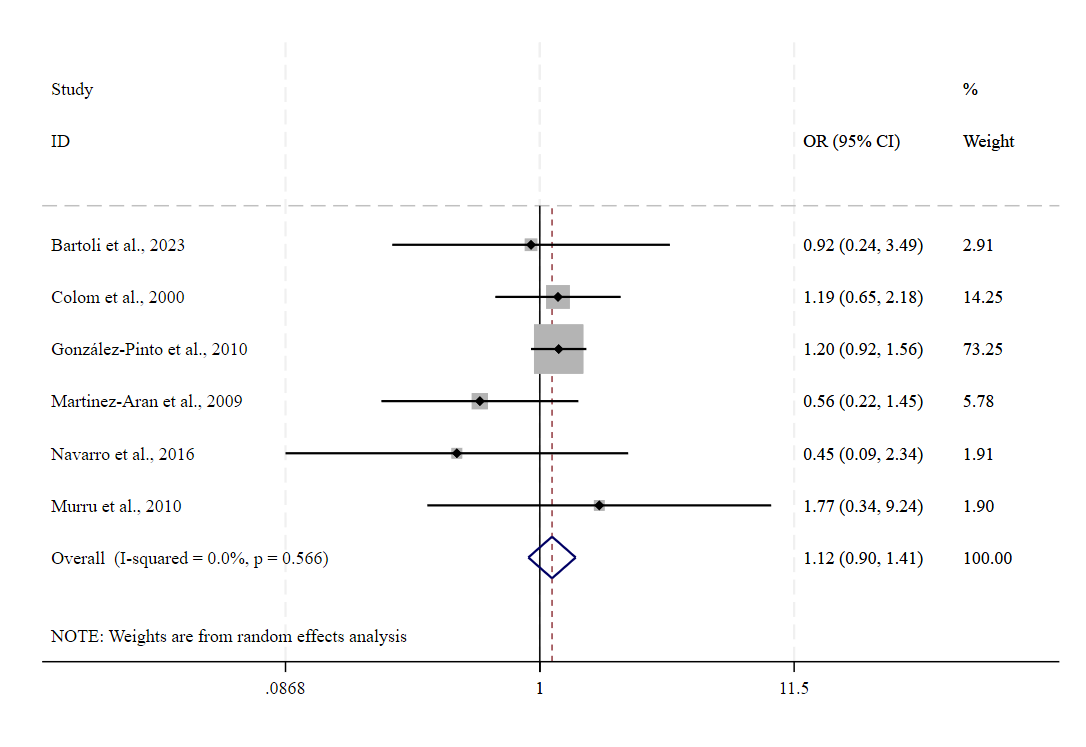


k=6; N=2,386; OR=1.13, 95%CI: 0.90 to 1.41, p=0.312; I²=0%

**Supplementary** **Figure 32.** Antipsychotic use in participants with poor adherence as compared with those with good adherence.


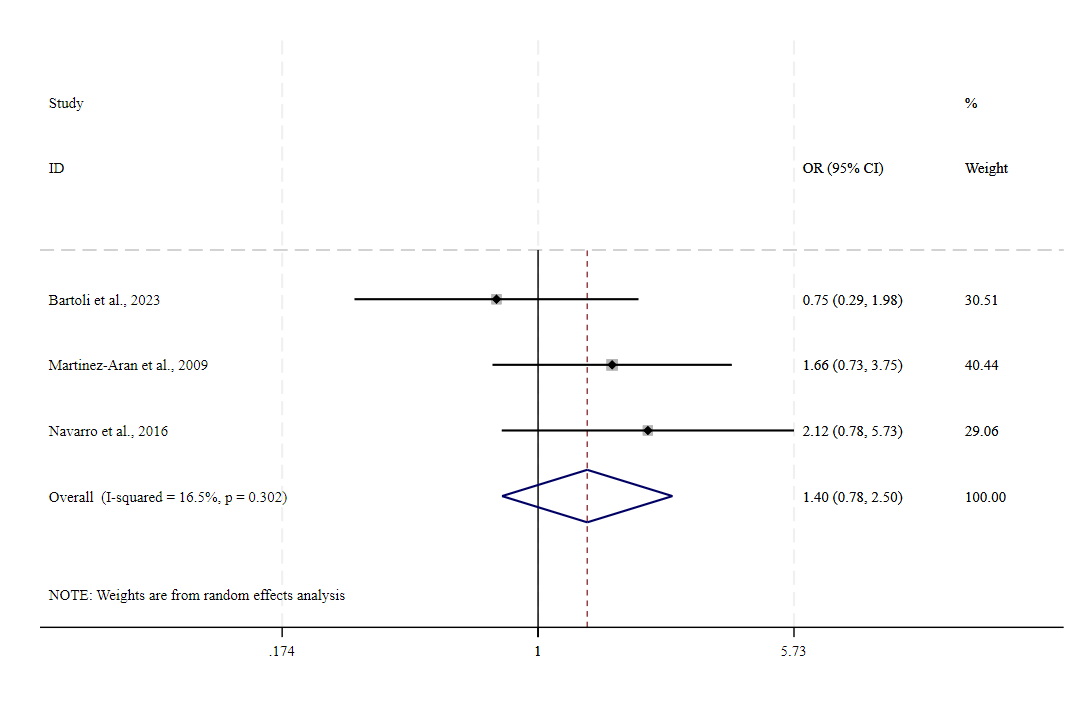


k=3; N=250; OR=1.40, 95%CI: 0.78 to 2.50, p=0.258; I²=16.5%

**Supplementary** **Figure 33.** First-generation antipsychotic use in participants with poor adherence as compared with those with good adherence.


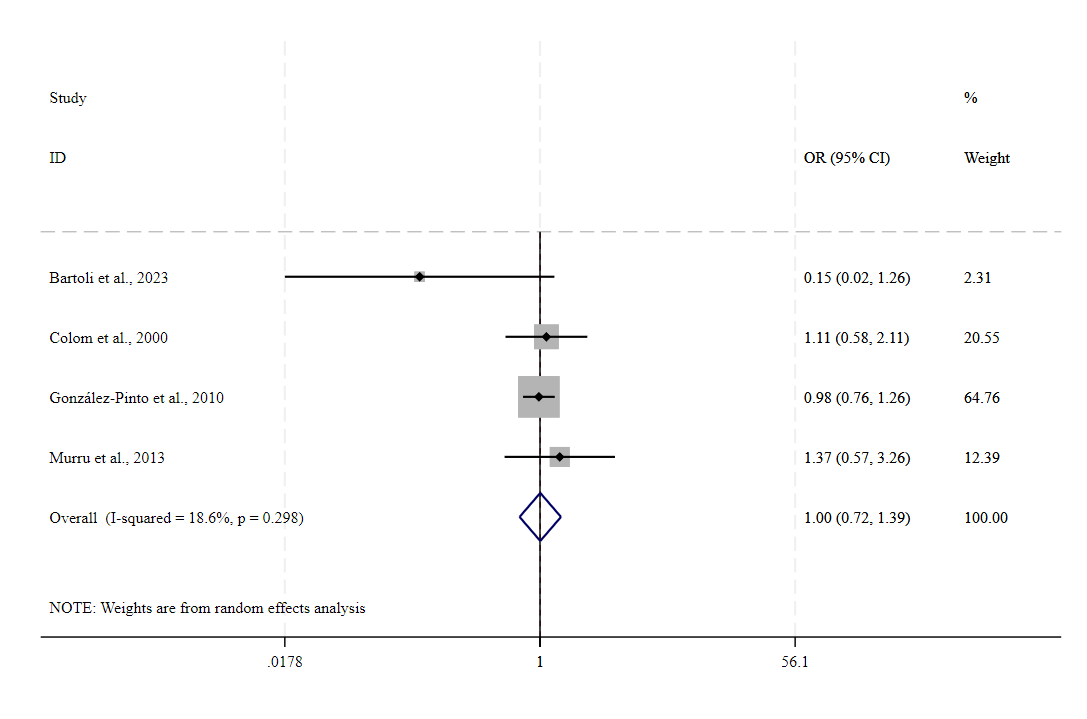


k=4; N=2,239; OR=1.00, 95%CI: 0.72 to 1.39, p=0.980; I²=18.6%

**Supplementary** **Figure 34.** Second-generation antipsychotic use in participants with poor adherence as compared with those with good adherence.


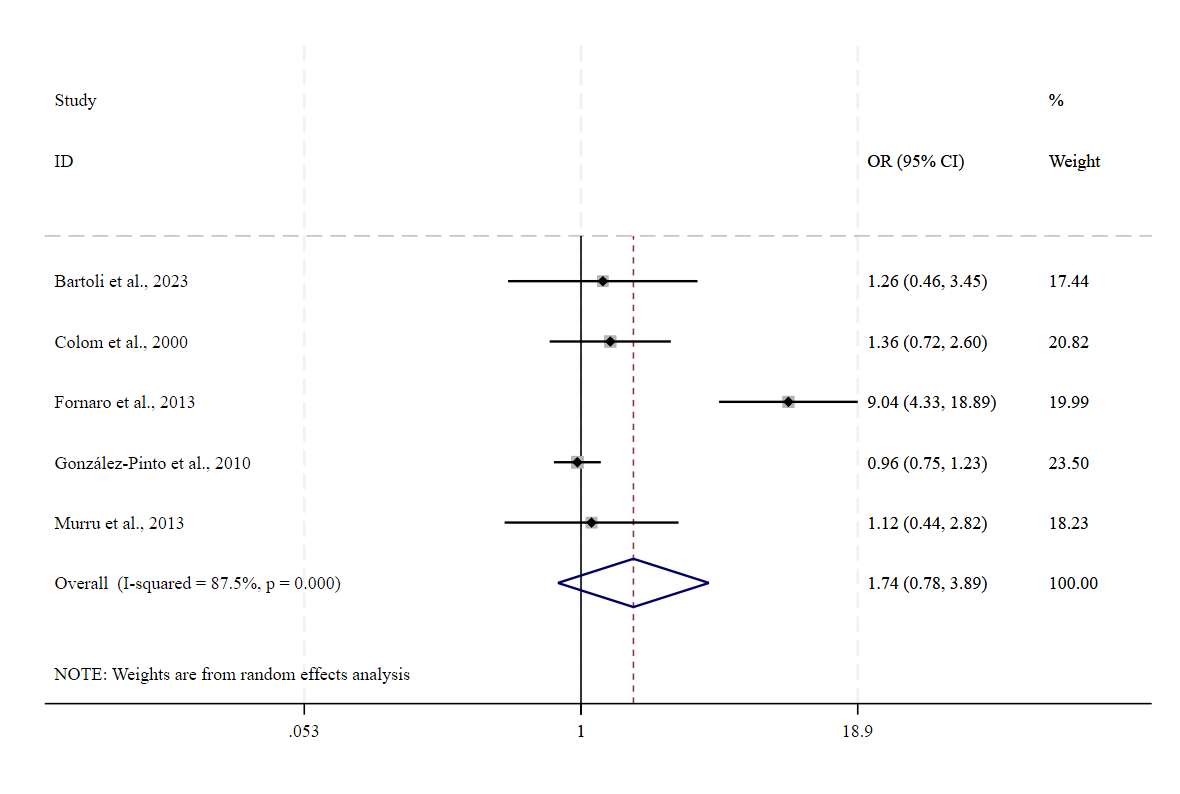


k=5; N=2,463; OR=1.75, 95%CI: 0.78 to 3.89, p=0.174; I²=87.5%

**Supplementary** **Figure 35.** Global severity scores in participants with poor adherence as compared with those with good adherence.


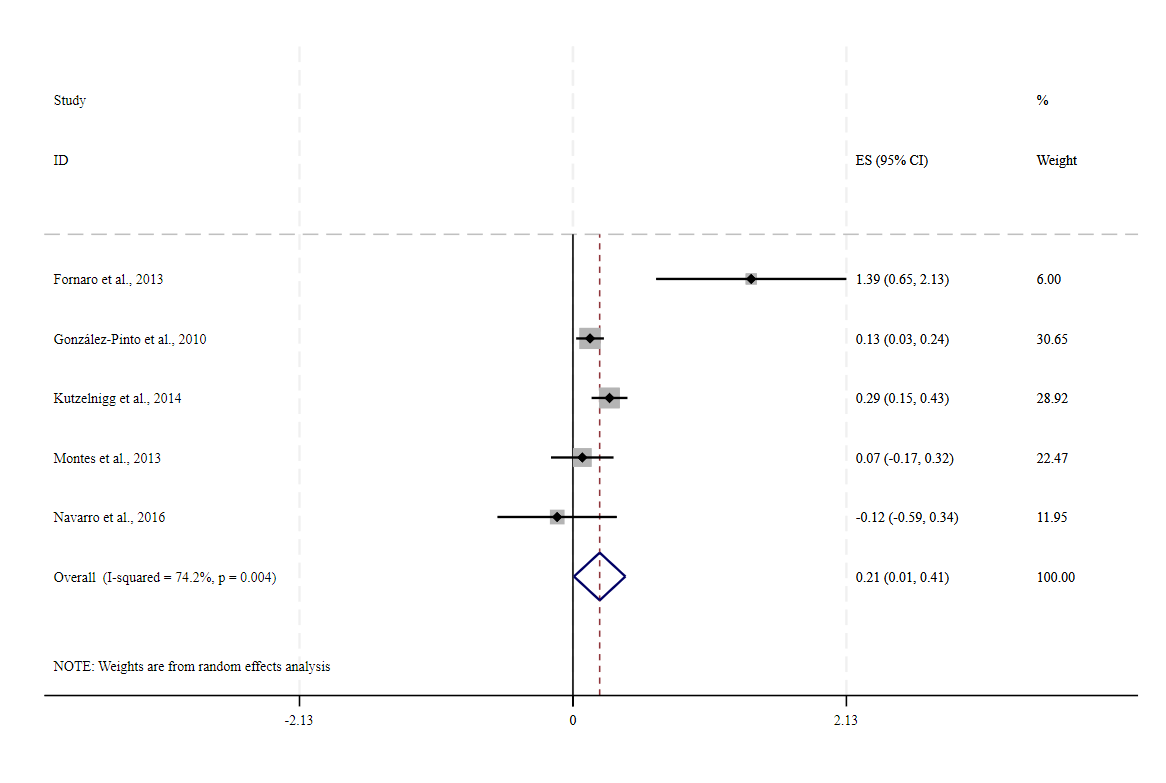


k=5; N=3,321; SMD=0.21, 95%CI: 0.01 to 0.41, p=0.042; I²=74.2%

**Supplementary** **Figure 36.** Insight scores in participants with poor adherence as compared with those with good adherence.


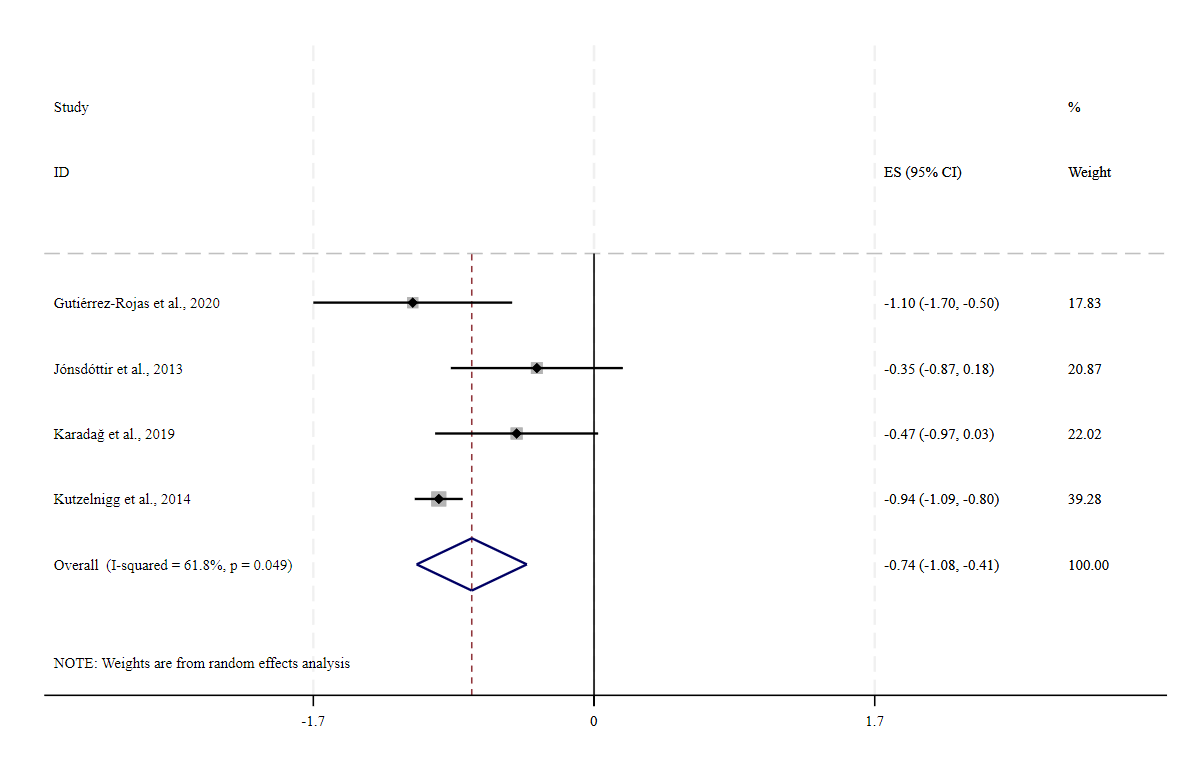


k=4; N=1,219; SMD=–0.74, 95%CI: –1.08 to –0.41, p<0.001; I²=61.8%

**Supplementary** **Figure 37.** Global functioning scores in participants with poor adherence as compared with those with good adherence.


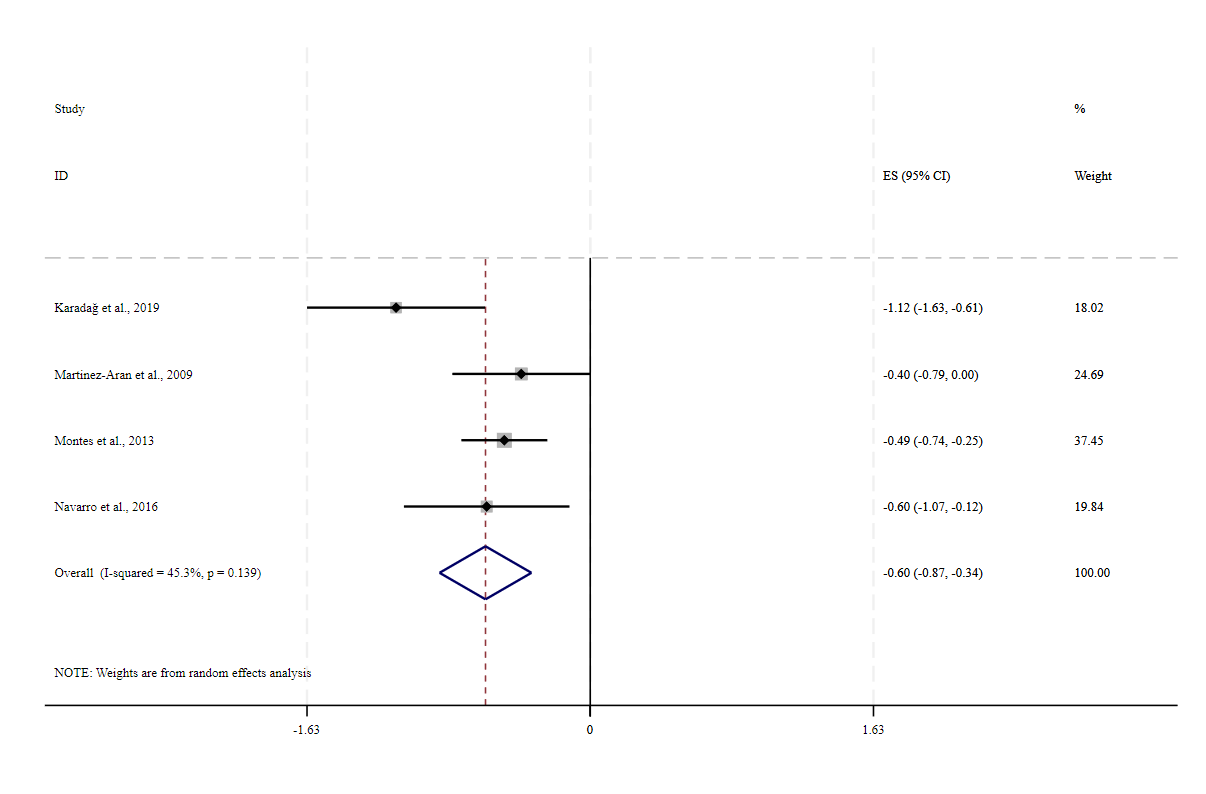


k=4; N=599; SMD=–0.60, 95%CI: –0.87 to –0.34, p<0.001; I²=45.3%

**Supplementary** **Figure 38.** Presence of general medical comorbidities in participants with poor adherence as compared with those with good adherence.


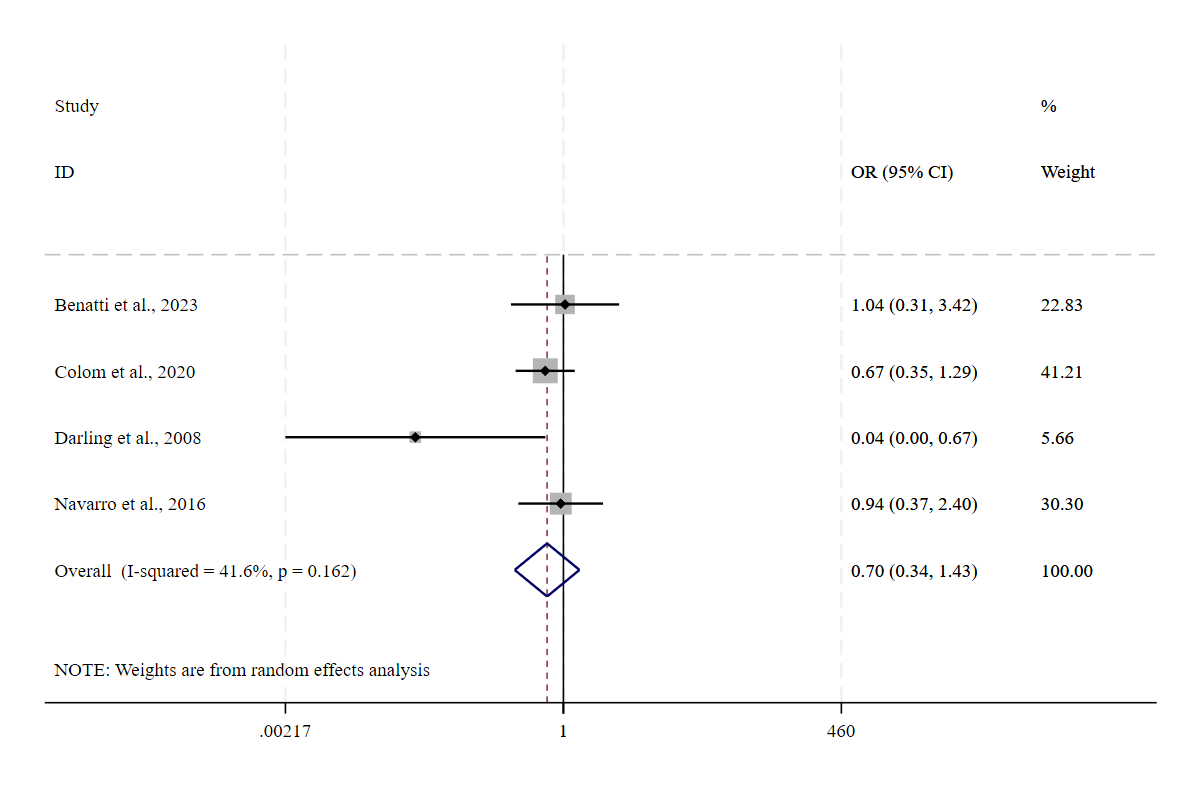


k=4; N=427; OR=0.70, 95%CI: 0.34 to 1.43, p=0.324; I²=41.6%

**Supplementary** **Figure 39.** Drug attitude scores in participants with poor adherence as compared with those with good adherence.


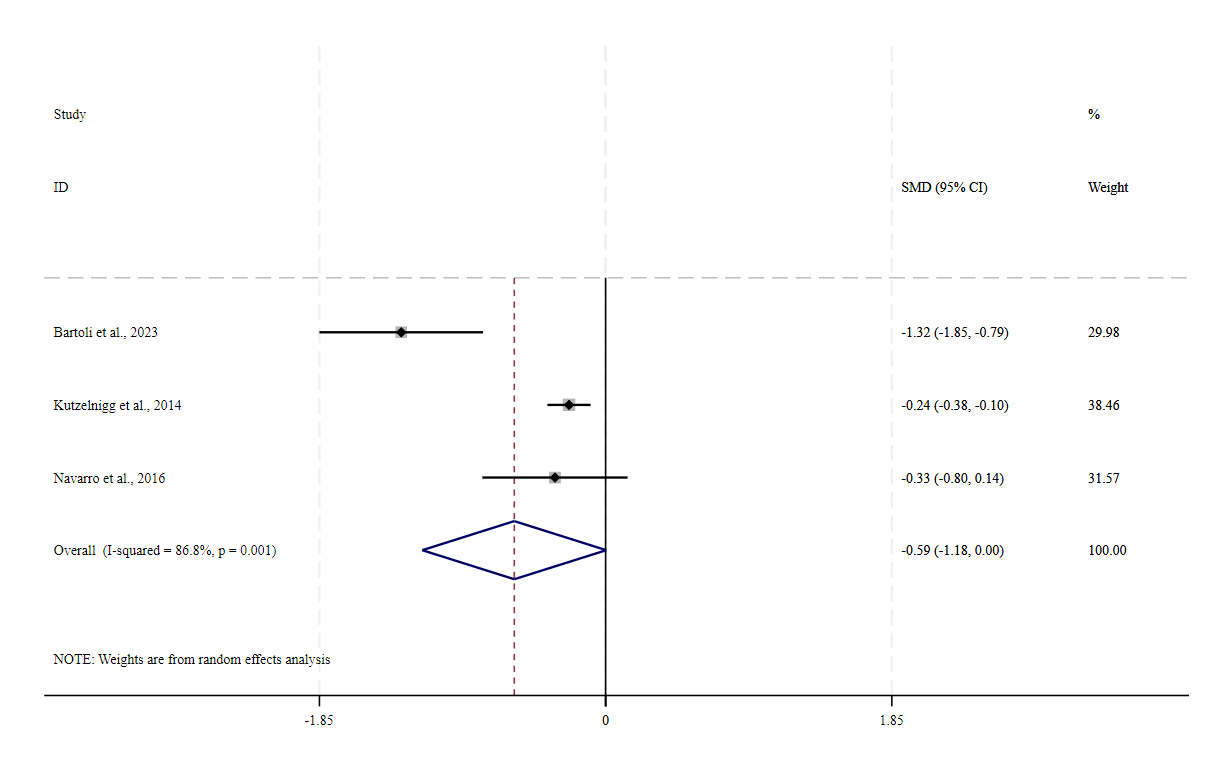


k=3; N=1,038; SMD=–0.59, 95%CI: –1.19 to 0.00, p=0.052; I²=86.8%
